# Genomic Diversity and Virulence Potential of ESBL- and AmpC-β-Lactamase-Producing *Escherichia coli* Strains From Healthy Food Animals Across Europe

**DOI:** 10.3389/fmicb.2021.626774

**Published:** 2021-04-01

**Authors:** Christa Ewers, Anno de Jong, Ellen Prenger-Berninghoff, Farid El Garch, Ursula Leidner, Sumeet K. Tiwari, Torsten Semmler

**Affiliations:** ^1^Department of Veterinary Medicine, Institute of Hygiene and Infectious Diseases of Animals, Justus Liebig University Giessen, Giessen, Germany; ^2^European Antimicrobial Susceptibility Surveillance in Animals (EASSA) Study Group, Executive Animal Health Study Center (CEESA), Brussels, Belgium; ^3^NG1 Microbial Genomics, Robert Koch Institute, Berlin, Germany

**Keywords:** *Escherichia coli*, livestock, ESBL, AmpC, virulence, sequence type, plasmid, pathotype

## Abstract

The role of livestock animals as a putative source of ESBL/pAmpC *E. coli* for humans is a central issue of research. In a large-scale pan-European surveillance, 2,993 commensal *Escherichia* spp. isolates were recovered from randomly collected fecal samples of healthy cattle, pigs and chickens in various abattoirs. One-hundred *Escherichia* spp. isolates (0.5% from cattle, 1.3% pigs, 8.0% chickens) fulfilled the criteria for cefotaxime and ceftazidime non-wildtype (EUCAST). *In silico* screening of WGS data of 99 isolates (98 *E. coli* and 1 *E. fergusonii*) revealed *bla*_SHV__–__12_ (32.3%), *bla*_CTX__–__M__–__1_ (24.2%), and *bla*_CMY__–__2_ (22.2%) as predominant ESBL/pAmpC types. Other types were *bla*_SHV__–__2_ (1.0%), *bla*_CTX__–__M__–__2__/__–__14__/__–__15_ (1.0/6.1/1.0%), and *bla*_TEM__–__52_ (5.1%). Six isolates revealed AmpC-promoter mutations (position −42 (C > T) and one carried *mcr-1*. The majority (91.3%) of ESBL/pAmpC genes were located on plasmids. SHV-12 was mainly (50%) encoded on IncI1α plasmids (pST-3/-26/-95), followed by IncX3 (12.5%) and IncK2 (3.1%). The *bla*_TEM__–__52_ genes were located on IncI1α-pST-36 (60%) and IncX1 plasmids (20%). The dominant plasmid lineage among CTX-M-1 isolates was IncI1α (pST-3/-295/-317) (87.5%), followed by IncN-pST-1 (8.3%). CMY-2 was mostly identified on IncI1α (pST-12/-2) (54.5%) and IncK2 (31.8%) plasmids. Several plasmids revealed high similarity to published plasmids from human and animal Enterobacteriaceae. The isolates were assigned to phylogroups A/C (34.7/7.1%), B1 (27.6%), B2 (3.1%), D/F (9.2/10.2%), E (5.1%), and to *E*. clades (3.0%). With 51 known and 2 novel MLST types, a wide variety of STs was found, including STs previously observed in human isolates (ST10/38/117/131/648). ESBL/AmpC types or STs were rarely correlated with the geographic origin of the isolates or animal species. Virulence gene typing identified extraintestinal pathogenic *E. coli* (ExPEC; 2.0%), avian pathogenic *E. coli* (APEC; 51.5%), and atypical enteropathogenic *E. coli* (EPEC; 6.1%). In conclusion, the high diversity of STs and phylogenetic groups provides hardly any hint for clonal spread of single lineages but hints toward the dissemination of cephalosporin resistance genes in livestock via distinct, globally successful plasmid lineages. Even though a number of isolates could not be assigned to a distinct pathotype, our finding of combined multidrug-resistance and virulence in this facultative pathogen should be considered an additional threat to public health.

## Introduction

Since the turn of the century, the prevalence of infections due to extended-spectrum cephalosporin-resistant (ESC-R) Enterobacteriaceae increased globally both in hospitals and in the community, entailing a major public health concern. At the same time, the prevalence of ESC-R *Escherichia coli* has been increasingly reported in livestock, in the food chain, and in companion animals (Ewers et al., 2012; Kaesbohrer et al., 2019). Consequently, there has been a controversial discussion about the role of animals as a putative source of ESC-R *E. coli* for humans either by direct contact or consumption of contaminated food for many years (Carattoli, 2008; Ewers et al., 2012).

Resistance to third and fourth generation cephalosporins, which are rated highest priority critically important antimicrobials as defined by the World Health Organisation (WHO, 2019), is frequently conferred by the hydrolytic activity of extended-spectrum β-lactamases (ESBL) and plasmid-mediated AmpC-β-lactamases (pAmpC). The most clinically significant ESBL variants belong to the CTX-M, TEM and SHV families, pAmpCs are mostly represented by the CMY family (Bush and Fisher, 2011; Ewers et al., 2012; Carattoli, 2013). The frequent localization of *bla*_ESBL_ and *bla*_AmpC_ genes on plasmids and their common association with mobile genetic elements, like transposons and insertions sequences, contributes to their successful spread. Horizontal transfer facilitates easy transmission of ESBL/pAmpCs between bacteria of the same or closely related species, including commensals and pathogens, and including the intestinal microbiota of animals and humans (Ewers et al., 2012; Carattoli, 2013; Liebana et al., 2013; Madec et al., 2017; Rozwandowicz et al., 2018). ESBL-/pAmpC-encoding plasmids determined in the European Union (EU) frequently belong to the incompatibility groups (Inc) F, A/C, N, HI2, I1, and K, which differ in their host range and self-transmissibility (EFSA Panel on Biological Hazards (BIOHAZ), 2011; Liakopoulos et al., 2016). To understand the spread of ESC-R *E. coli* it is not only essential to consider the genetic context of ESBL/pAmpC plasmids but also the phylogenetic epidemiology and pathogenic potential of the bacterium.

*E. coli* comprises non-pathogenic commensals and strains that cause a variety of diseases in humans and animals. *E. coli* strains capable of causing extraintestinal infections, such as urinary tract infection, blood stream infection and soft tissue damage, are termed extraintestinal pathogenic *E. coli* (ExPEC), whereas strains that lead to intestinal diseases are designated as intestinal pathogenic *E. coli* (InPEC). While the classification of InPEC into different pathovars follows defined genetic criteria, i.e., concrete virulence-associated genes (VAGs) and/or phenotypic features, a wide range of VAGs have been associated with ExPEC and its various pathovars. It is assumed that ExPEC reside in the normal gut microbiota of healthy mammals and birds (Ewers et al., 2009; Koehler and Dobrindt, 2011; Starcic Erjavec and Zgur-Bertok, 2015). The combination of antimicrobial resistance (AMR) and virulence in *E. coli* strains constitutes a significant public health risk as exemplified by the successful global dissemination of ESBL-ExPEC lineages ST131 und ST648 (Ewers et al., 2014a; Schaufler et al., 2019), or by the CTX-M-15 producing enteroaggregative and Shigatoxin producing *E. coli* O104:H4 strains that caused the large German EHEC outbreak in 2011 (Karch et al., 2012).

In this study, we determined the prevalence of ESC-non-susceptible (ESC-non-S) and ESC-R isolates in a European collection of presumptive ESBL/pAmpC-producing *E. coli* and characterized ESBL/pAmpC plasmids and AMR genes as well as the clonal diversity and serotype of the strains based on whole genome sequence (WGS) analysis. Virulence gene profiles were determined to facilitate classification into ExPEC and InPEC pathovars in order to assess the pathogenic potential of antimicrobial resistant strains.

## Materials and Methods

### Bacterial Isolates

The European Antimicrobial Susceptibility Surveillance in Animals (EASSA) monitors the antimicrobial susceptibility of zoonotic and commensal bacteria in healthy food-producing animals at slaughter across Europe (de Jong et al., 2013). The EASSA project includes the major countries of production of beef cattle, slaughter pigs and broiler chickens in the EU. Five or six countries were selected per animal species with ≥4 slaughterhouses in each country providing samples (usually about 200 per animal species). Colon and cecal samples were randomly collected from each of the major food-producing animal species. At most one isolate of each bacterial species was retained from each animal, which was randomly selected as being representative of a whole herd or flock. Our study comprised 2,993 non-repetitive *E. coli* isolates from broiler chicken (*n* = 1016), cattle (*n* = 841), pig (*n* = 1136) isolated in Belgium (*n* = 167), Denmark (*n* = 208), France (*n* = 588), Germany (*n* = 426), Hungary (*n* = 328), Poland (*n* = 182), Spain (*n* = 410), the Netherlands (*n* = 387), and the United Kingdom (*n* = 297) isolated from February 2013 to March 2015 (2013, 1,181 isolates; 2014, 1,588 isolates; 2015, 224 isolates).

### Antimicrobial Susceptibility Testing and Verification of Bacterial Species

Of the total *Escherichia* spp. collection 100 isolates fulfilled the criteria for cefotaxime and ceftazidime non-wild type according to EUCAST criteria, i.e., reduced susceptible or resistant to one or both cephalosporins [isolates with MICs of cefotaxime and/or ceftazidime ≥ 1 mg/L; EUCAST (The European Committee on Antimicrobial Susceptibility Testing), 2015; Table 1]. MALDI-TOF MS analysis (Bruker Daltonics, Bremen, Germany) confirmed 99 of these isolates as *E. coli* and one isolate as *E. fergusonii*.

**TABLE 1:** ESBL/pAmpC types in ESC-R *E. coli* (*n* = 99) and *E. fergusonii* (*n* = 1) from the EASSA collection 2013–2015.

		**Ceftazidime**		**Cefotaxime**		**Country^*b*^**									**Number of isolates**		
						**(number of isolates)**									**per animal species^*c*^**		
**ESBL/AmpC**	**No. of isolates**	**MIC range (mg/L)**	**MIC_50_ (mg/L)^*a*^**	**MIC range (mg/L)**	**MIC_50_ (mg/L)^*a*^**	**BE**	**DE**	**DK**	**ES**	**FR**	**HU**	**NL**	**PL**	**UK**	**Chicken**	**Pig**	**Cattle**
SHV-12	31	8–128	64	0.5–32	2				25	1	2	3			28	3	
SHV-2	1	2	−	4	−				1						1		
TEM-52	5	8–32	−	4–16	−				2		2	1			4	1	
CTX-M-1	23	2–16	8	8– > 32	> 32		4		3	10	5			1	17	5	1
CTX-M-2	1	4	−	32	−	1											1
CTX-M-14	6	0.5–64	−	8– > 32	−				6						4	2	
CTX-M-15	1	16	−	> 32	−								1				1
CMY-2	20	16–128	32	4–16	8	1			6		11	2			18	1	1
SHV-12 & CMY-2	1	128	−	16	−						1				1		
CTX-M-1 & CMY-2	1	64	−	8	−					1					1		
none/n.d.	9/1	8–32	−	2–32	−		1		3		2	4			7	3	
**Totals, if applicable**	**100**	**0.5–128**	**32**	**0.5– > 32**	**8**	**2**	**5**	**0**	**46**	**12**	**23**	**10**	**1**	**1**	**81**	**15**	**4**

*n.d.: not determined (no WGS data; the relative proportion of ESBL/AmpC types among isolates given in the text refers to 99 isolates). ^*a*^MIC_50_ was calculated if number of isolates exceeded 10. ^*b*^Country abbreviations are Belgium (BE), Germany (DE), Denmark (DK), Spain (ES), France (FR), Hungary (HU), the Netherlands (NL), Poland (PL), United Kingdom (UK). ^*c*^Chicken specimens were collected in ES, FR, HU, NL, UK; pig specimens in DE, DK, ES, FR, NL, UK; cattle specimens in BE, DE, FR, PL, and UK.*

The 100 isolates were further investigated for their susceptibility to 16 antimicrobials/antimicrobial combinations comprising 12 antibiotic classes used in human and/or veterinary medicine including the β-lactam antimicrobials ampicillin, cefepime, cefotaxime, ceftazidime and cefoxitin by using agar dilution according to Clinical Laboratory Standard Institute standards (CLSI, 2013). Minimum inhibitory concentrations (MICs) were interpreted according to CLSI criteria (CLSI, 2018), except those for colistin and tigecycline which were interpreted according to EUCAST guidelines [EUCAST (The European Committee on Antimicrobial Susceptibility Testing), 2015]. The *E. coli* strain ATCC 25922 was used for quality control. Multi-drug resistance (MDR) of an isolate was defined as clinical resistance to at least one agent in three or more antimicrobial classes (Magiorakos et al., 2012). The antimicrobials (and classes) included were ampicillin (penicillins), cefoxitin (cephamycins), cefotaxime, ceftazidime, and cefepime (extended-spectrum cephalosporins), ciprofloxacin (fluoroquinolones), chloramphenicol (phenicols), colistin (polymyxins), gentamicin (aminoglycosides), meropenem (carbapenems), trimethoprim/sulfamethoxazole (folate pathway inhibitors), sulfisoxazole (sulfonamides), tetracycline (tetracyclines), and tigecycline (glycylcyclines).

### Whole Genome Sequencing

Ninety-nine cefotaxime and ceftazidime non-wild type *Escherichia* spp. isolates (one isolate was excluded due to repeated DNA degradation) were whole genome sequenced. DNA was extracted from the isolates using the DNA Blood and Tissue Kit according to the manufacturer‘s instruction (Qiagen, Hilden, Germany), followed by library preparation, using Nextera XT library (Illumina, San Diego, United States). DNA was sequenced by using an Illumina NextSeq 550 with multiplexing of 70 samples per flow cell using 150 bp paired end reads and a minimum of 70-fold coverage. Raw reads were adapter-trimmed by Flexbar v.3.0.3 (Resource Identification Portal RRID: SCR_013001), corrected using BayesHammer and assembled *de novo* using SPAdes v3.12.1 (RRID: SCR_000131). Assembled draft genomes were annotated using Prodigal (Prodigal, RRID: SCR_011936).

### Resistance Gene Screening and Integron Typing

The web-based tool ResFinder 3.2^1^, hosted at the Center for Genomic Epidemiology (CGE), was used to identify resistance genes and chromosomal mutations related to β-lactam (*ampC* promoter mutation), fluoroquinolone (mutations in *gyrA*, *gyrB*, *parA*, and *parC*), and colistin resistance (*pmrAB*) based on WGS (Zankari et al., 2012). Resistance gene screening was carried out by BLASTn (90% identity and 90% query coverage) analysis against homologous genes present in the Comprehensive Antibiotic Resistance Database^2^.

To investigate the distribution of class 1, class 2, and class 3 integrons with prevalent arrays in the genomes of our *Escherichia* spp. isolates, we performed BLAST search against the available databases GenBank and INTEGRALL 1.2, using filter parameters of more than 90% nucleotide identity and at least 80% query coverage.

### Plasmid Analysis

PlasmidFinder 2.1 and pMLST 2.0 (Carattoli et al., 2014) were applied to determine plasmid replicon types and plasmid multilocus sequence types (pST). To further characterize the identified ESBL and pAmpC gene harboring contigs, all contigs were aligned using Geneious (v. 8.1.9, Biomatters Ltd., Auckland, New Zealand) (Geneious, RRID: SCR_010519) to the respective genes (ESBL genes: *bla*_CTX__–__M__–__1_ gene, GenBank accession no. DQ915955.1; *bla*_CTX__–__M__–__2_, EU622041.1; *bla*_CTX__–__M__–__14_, AF252622.2; *bla*_CTX__–__M__–__15_, DQ302097.1; *bla*_SHV__–__2_, AF282921.1; *bla*_SHV__–__12_, AF462395.1; *bla*_TEM__–__52_, AF027199.1; pAmpC gene: *bla*_CMY__–__2_, X91840.1). Contigs containing one of these genes were aligned to publicly available ESBL-/pAmpC plasmids of different incompatibility groups (Inc) from GenBank (GenBank, RRID: SCR_002760) (Supplementary Table S1). All contigs of a respective isolate were then aligned to the reference plasmid that revealed highest similarity. In addition, contigs were mapped to the selected reference plasmids using the Geneious Map to Reference (Geneious, RRID: SCR_010519). *In silico* constructed plasmids were further examined for mobile genetic elements, using ISfinder (RRID: SCR_003020).

To display circular comparisons between plasmids we used the blast ring image generator software BRIG Version 0.95 (RRID: SCR_007802).

To test whether ESBL and AmpC-β-lactamase genes were transferable, conjugation was performed by the broth filter mating method at 37°C on 40 isolates which represented different ESBL/pAmpC genes and plasmid replicon types using plasmid-free sodium azide resistant *E. coli* J53 (J53 Azi^*R*^) as recipient. Transconjugants were selected on Mueller Hinton agar plates supplemented with 100 mg/L sodium azide and 4 mg/L cefotaxime (Sigma-Aldrich, Germany). To confirm successful plasmid transfer, antimicrobial susceptibility testing of transconjugants and PCR for ESBL/pAmpC genes were performed. In addition to WGS analysis, plasmid sizes and location of ESBL/pAmpC genes were determined for all 99 isolates by S1 nuclease-restriction followed by pulsed-field gel electrophoresis and subsequent southern blotting. To verify the genomic location of ESBL and pAmpC genes we further performed an *in silico* search of whole genome sequences with mlplasmids v. 1.0.0 (Arredondo-Alonso et al., 2018).

### Phylogenetic Analysis

Phylogenetic groups were determined by using the ClermonTyping method and its associated web-interface ClermonTyper, that allows a given strain sequence to be assigned to *E. albertii, E. fergusonii*, *Escherichia* clades I-V, *E. coli sensu stricto* as well as to the seven main *E. coli* phylogroups A, B1, C, E, D, F, and B2 (Clermont et al., 2013; Beghain et al., 2018). MLST 2.0^3^ (Larsen et al., 2012) was applied to identify the multilocus sequence type (RRID: SCR_010245) of *E. coli* isolates following the Achtman scheme^4^, which represents a 7-gene-scheme including genes *adk*, *fumC*, *gyrB*, *icd*, *mdh*, *purA*, and *recA*.

### Detection of Serotype and Virulence-Associated Genes

The sero(geno)type of *E. coli* strains was determined using the web-based serotyping tool SerotypeFinder 2.0^5^ (Joensen et al., 2015). Screening for VAGs was carried out by NCBI BLASTn (RRID: SCR_004870) analysis against homologous genes present in an in-house database of 800 VAGs, gene variants or genomic islands from a subset of the VirulenceFinder database and in-house created and manually curated VAG reference sequences. Coverage length and sequence identity thresholds were 80 and 90%. We searched for genes that were previously linked with intestinal and extraintestinal pathogenic *E. coli* pathovars and that belonged to different categories (adhesin, toxin, iron uptake system, capsule synthesis, auto-transporter, and secretions system). A VAG score was definded as the total number of VAGs within an isolate. In case a VAG was detected multiple times within a single isolate, it was only counted once. The *kpsM*, *afa/dra*, and *sfa/foc* operons were considered present if any of the corresponding genes or allelic variants were identified. All *E. coli* isolates were further analyzed for the presence of *fimH* gene and allele type by aligning to a FimH database using FimTyper 1.0^6^.

### Determination of Associations Between *E. coli* Host, Country of Origin, Phylogroups, VAGs, and AMR Genes

The clustering of binary matrix indicating the presence and absence of antibiotic and virulence classes was performed by using package Rtsne version 0.15 (RRID: SCR_016900) with 5000 iteration and perplexity 15 in R version 3.6.1 (R Project for Statistical Computing, RRID: SCR_001905). Microreact version 5.93.0 was applied to visualize the clustering pattern in association with the metadata of the strains (Argimon et al., 2016). Kruskal-Wallis test as well as χ^2^ and Fisher’s exact test (IBM SPSS Statistics 27) were used to determine whether proportions for one variable (AMR gene, VAGs) were different between phylogroups and isolate origin (host and country). *P*-values < 0.001 were considered as statistical significant.

### Core Genome Analysis

Phylogenetic and population genetic relationships were determined by applying a gene-by-gene approach on the dataset to generate a core genome alignment and subsequently a phylogenetic tree. The core genome alignment was assembled by a gene-wise alignment with Mafft v7.407 (RRID: SCR_011811), of 1,366 core genes that were present in at least 99% of the strains (sequence similarity min. 70%, sequence coverage min. 90%) and were concatenated afterward. The resulting alignment was used to infer a phylogeny with 100 bootstrap replicates using RAxML v.8.2.10 (RAxML, RRID: SCR_006086) with a General Time Reversible model and gamma correction for among site rate variation. iTOL v5 (RRID: SCR_018174) was used to visualize the population structure in the context of available metadata.

## Results

### Antimicrobial Susceptibility

Of the total collection of 2,993 presumed *E. coli* isolates, 100 (3.3%) isolates fulfilled the criteria for cefotaxime and ceftazidime non-wild type, i.e., reduced susceptible (ESC-non-S) or resistant (ESC-R) to one or both cephalosporins [EUCAST (The European Committee on Antimicrobial Susceptibility Testing), 2015; CLSI, 2018]. Spain (46 out of 100 ESC-non-S/ESC-R isolates; 46/410 = 11.2% within-country rate) and Hungary (23/328 = 7.0%) were the main sources of ESC-non-S/ESC-R isolates followed by France (12/588 = 2.0%), the Netherlands (10/387 = 2.6%), Germany (5/426 = 1.2%), Belgium (2/167 = 1.2%), Poland (1/182 = 0.6%), and United Kingdom (1/297 = 0.3%) while no ESC-non-S/ESC-R isolate was retrieved from 208 *E. coli* strains from Denmark. Most of the ESC-non-S/ESC-R isolates originated from poultry (81/100 = 81%), followed by pigs (15/100 = 15%), and cattle (4/100 = 4%) (Table 1). Also with regard to the total number of isolates investigated, ESC-non-S/ESC-R isolates were more prevalent in chickens (*n* = 81/1016; 8.0%) compared with pigs (*n* = 15/1136; 1.3%) and cattle (*n* = 4/841; 0.5%).

Percentages of clinical resistance of 100 ESC-non-S/ESC-R *Escherichia* spp. isolates to compounds other than cefotaxime and ceftazidime were in decreasing number: ampicillin 100%, sulfisoxazole 78%, tetracycline 68%, nalidixic acid 67%, trimethoprim/sulfamethoxazole 37%, ciprofloxacin 32%, cefoxitin 27%, chloramphenicol 27%, gentamicin 4%, and colistin 1%. All strains were susceptible to meropenem and tigecycline.

Almost all isolates (97%) revealed MDR. The most frequent MDR phenotype was combined resistance to penicillins, cephalosporins, tetracycline, sulfisoxazole, and either phenicols or folate pathway inhibitors (*n* = 8 each). Different proportions of isolates showed resistance to two (3%), three (18%), four (27%), five (23%), six (19%), seven (7%), and eight (3%) antibiotic classes. On average, isolates from cattle, chickens and pigs revealed a mean number of resistance to 4.3, 3.5, and 3.0 antibiotic classes, respectively. Thirty-four phenotypic AMR patterns (based on resistance of isolates to 12 antibiotic classes) were distinguished (Supplementary Table S2). Eleven of these patterns were unique; the remaining patterns were dispersed among a number of 2–8 isolates. AMR patterns appeared to be unrelated to host and country origin of the *E. coli* isolates.

### Antimicrobial Resistance Genes and Integrons

The genomes of 99 out of 100 ESC-non-S/ESC-R isolates were sequenced successfully. One isolate from a chicken in Spain turned out to belong to the species *E. fergusonii*. Ninety out of these 99 isolates (90.9%) possessed one or more ESBL/pAmpC genes (Table 1). Another six isolates that lacked ESBL/pAmpC genes revealed mutations in the promoter of the chromosomally encoded *ampC* gene when compared with the wildtype *E. coli* K12 sequence (ATCC 25922; NZ_CP009072.1). Mutations were located at positions −42 (alternate −35 box, C > T), −18 (alternate −10 box, G > A), + 81 (AmpC coding region, A > G), and outside functional promtoter elements and AmpC coding region (−1, C > T; + 58, C > T). In three ESBL- and AmpC negative isolates, namely IHIT31981, IHIT31985 (both from chicken in the Netherlands) and IHIT32069 (pig, Netherlands), the genetic background of the ESC-R phenotype remained unclear.

**FIGURE 1:**
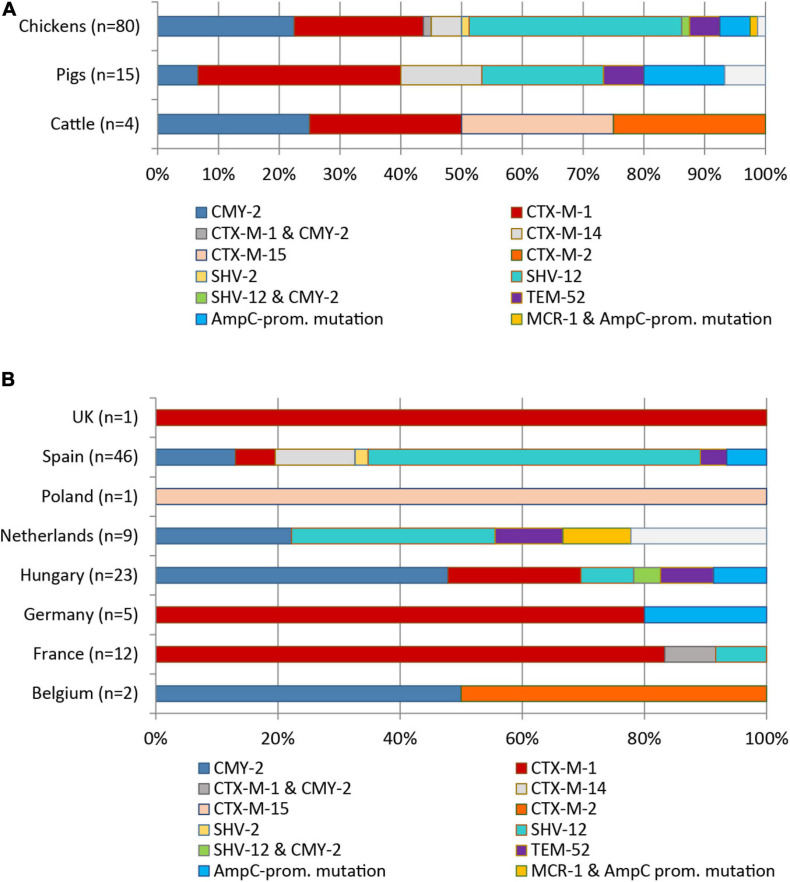
Distribution of ESBL/pAmpC genes and AmpC promotor mutations among 99 ESC-non-S/ESC-R *E. coli* isolates with regard to livestock species **(A)** and country of origin **(B)**.

Eight different ESBL/pAmpC genes were detected among the 99 *Escherichia* spp. isolates (98 *E. coli*, one *E. fergusonii*) from chickens (*n* = 80), pigs (*n* = 15), and cattle (*n* = 4) (Figure 1A). *Bla*_SHV__–__12_ was the most prevalent ESBL gene (32.3%) with the highest frequency observed among isolates from chickens (29/80 = 36.3%), followed by swine (3/15 = 20.0%). With regard to the geographical origin, isolates from Spain (25/46 = 54.3%) and from the Netherlands (3/9 = 33.3%) most frequently harbored *bla*_SHV__–__12_ (Figure 1B).

The genes *bla*_CTX__–__M__–__1_ (24/99, 24.2%) and *bla*_CMY__–__2_ (22/99, 22.2%) were the second and third most identified ESBL/pAmpC genes among the 99 *Escherichia* spp. isolates. The genes were nearly equally present among isolates from chickens (*bla*_CTX__–__M__–__1_: 18/80 = 22.5%; *bla*_CMY__–__2_: 20/80 = 25.0%). The *bla*_CTX__–__M__–__1_ gene showed the highest frequency in ESC-non-S/ESC-R isolates from pigs (33.3%), while with 25.0% of the four cattle isolates harbored this gene. With respect to country origin, the CTX-M-1 encoding gene was predominant in isolates from France (11/12 = 91.7%) while it was the only ESBL gene detected among isolates from Germany and the United Kingdom. Two isolates revealed co-presence of ESBL/pAmpC genes: *bla*_CMY__–__2_ and *bla*_SHV__–__12_ (chicken, Hungary) and *bla*_CMY__–__2_ and *bla*_CTX__–__M__–__1_ (chicken, France). Other ESBL types observed were *bla*_SHV__–__2_ (1/99 = 1.0%), *bla*_CTX__–__M__–__2__/__–__14__/__–__15_ (1, 6, and 1 out of 99 = 1.0, 6.1, and 1.0%), and *bla*_TEM__–__52_ (5/99 = 5.1%) (Figures 1A,B).

Besides ESBL/pAmpC genes, we observed several other genes among the 99 *Escherichia* spp. isolates. Additional β-lactamase genes identified were *bla*_TEM__–__1_ (32.3%) and *bla*_*OXA*__–__9_ (1.0%). Different proportions of isolates harbored genes encoding for resistance to aminoglycosides (*aadA1*, 43.4%; *aadA2*, 27.3%; *aadA5*, 11.1%; *aadA15* (2.0%); *aadA24* (2.0%), *aac(6‘)-Ib*, 2.0%; *strA/strB*, 28.3%; *aac(3)*-*IId*, 1.0%; *aac(3)IVa*, 4.0%; *aph(3′)-Ia*, 1.0%; *aph(3′)-Ic*, 3.0%), tetracyclines [*tet*(A), 52.5%; *tet*(B), 15.2%], folate pathway inhibitors (*dfrA1*, 24.2%; *dfrA7*, 1.0%; *dfrA8*, 1.0%; *dfrA12*, 4.0%; *dfrA16*, 1.0%; *dfrA17*, 12.1%; *sul1*, 24.2%; *sul2*, 44.4%; *sul3*, 25.3%), phenicols (*catA1*, 9.1%; *catB2* 1.0%; *catB3*, 2.2%; *cmlA*, 22.2%; *floR*, 2.0%), lincosamides [*Inu*(F), 2.0%], and macrolides [*mph*(A), 2.0%; *mph*(B), 9.1%, *mef*(B), 1.0%]. Apart from *tet*(A) and *mef*(B), the isolates commonly harbored other genes encoding efflux pumps and/or products involved in membrane permeability, such as *acrAB* (100%), *acrAD* (96.0%), *acrEF* (93.9%), *mdtAB* (98.0%), and *mdtEF* (98.0%). Almost 40% of the resistance plasmids revealed co-carriage of ESBL/pAmpC and AMR genes encoding tetracycline, aminoglycoside, phenicol, and fluoroquinolone resistance. Detailed results from resistance gene screening are provided in Supplementary Document S1A.

Aminoglycoside, trimethoprim and sulfonamide resistance genes were frequently located on integrons. More than half of the isolates harbored gene cassettes (GC) associated with integron class I (55.6%). Integron class II was less prevalent (3.0%) and integron class III was not present. The 58 integron-positive isolates revealed phenotypic resistance (mean value) to 5.5 antibiotic classes compared to 3.5 classes for isolates without integrons. In total, 17 and 2 different GCs were found on class I and class II integrons, respectively. The most common class I integron GC was *estX-psp-aadA2-cmlA1-aadA1*-*qacI* embedded in the genetic platform *tnp440*-*sul3*-*orf1*-*ygkA*-*yusZ*-*orf1*-?*mef(B)-tnp26* (7/55 integron class I isolates; 12.7%). It was almost identical with the GC present in SHV-12-IncI1α plasmid pCAZ590 (GenBank LT669764.1) from an *E. coli* isolate from chicken in Germany (Alonso et al., 2017). Other frequently identified class I integron GCs were *dfrA1-aadA1*-*qacEdelta1-sul1* (18.2%), and *dfrA17-aadA5-tnp-orf1-orf5-sul2* (14.6%). Eleven other GCs were distributed among the remaining 14 isolates, which is detailed in Supplementary Document S2.

10 isolates (10.1%) carried plasmid-mediated quinolone resistance (PMQR) gene *qnrS1*. Sixty-six strains (66.7%) had at least one mutation in GyrA [D87Y (*n* = 2), S83L (*n* = 33), S83L and D87H (*n* = 1), S83L and D87N (*n* = 29)], and 55.0% of these strains possessed at least one additional mutation in ParC [mostly S80I (*n* = 26)] (Supplementary Document S2). All isolates with wildtype GyrA and ParC sequences (*n* = 33) were phenotypically susceptible to ciprofloxacin, even though three of these isolates harbored the *qnrS1* gene (MICs 0.5–1.0 mg/L). On the other hand, resistance to ciprofloxacin was usually (97.7%) associated with mutations in *gyrA* and *parC* genes, irrespective of the presence of *qnrS1* in the isolates.

Furthermore, we identified the plasmid-mediated colistin resistance gene *mcr-1* in one colistin resistant strain isolated from a broiler chicken sample in the Netherlands in 2013. Other *mcr* variants (*mcr-2*–*mcr-10*) or alleles were not present.

### Genomic Location of ESBL, pAmpC and mcr-1 Genes and Plasmid Analysis

The most frequent replicon types identified were Col (MG828, RNAI, 156, pVC, 8282, BS512, MGD2, E10, MP18) (*n* = 161), FIB (*n* = 78), FII (*n* = 67), I1 (*n* = 65), FIC (*n* = 31), P0111 (*n* = 23), X1 (*n* = 20), and B/O/K/Z (*n* = 19), Q1 (*n* = 15), and FIA (*n* = 14). Among the replicon types that were found less than 10 times were I2 (*n* = 8), N (*n* = 6), X4 (*n* = 6), Y (*n* = 5), HI2 (*n* = 4), X3 (*n* = 4), and A/C2 (*n* = 2). Contig alignment to several completely assembled plasmids from GenBank database carrying the different ESBL-/AmpC/*mcr-1* genes identified in this study, together with mlplasmid analysis results and data from S1 nuclease digestion and Southern Blot hybridization enabled the identification of *bla*_*ESBL/pAmpC*_ and *mcr-1* gene location and calculation of plasmid sizes.

The majority of strains (83/91; 91.2%) carried their ESBL/pAmpC/MCR-1 genes on plasmids. For 30 isolates, there was a link between ESBL/pAmpC genes and replicon sequences on the same *de novo* assembled contig. In the remaining isolates, WGS-based reconstruction, i.e., a mapping of contigs to previously published plasmid sequences together with plasmid prediction of contigs by using mlplasmids was performed to deduce the respective plasmid contigs. In some cases, we could not reconstruct the entire plasmid backbones. Consequently, Inc types of ESBL/pAmpC encoding plasmids could either not (*n* = 3 isolates Inc not typable) or only partially (*n* = 7 isolates non-IncI1-type) be assigned Supplementary Document S2. Transconjugation assays with 40 selected strains confirmed the transferability of ESBL/pAmpC/MCR-1 plasmids and phenotypic resistance (beta-lactam or colistin) for all strains and transconjugants, respectively (data not shown).

#### CTX-M Type β-Lactamases

The most common gene-plasmid combination was IncI1α-*bla*_CTX__–__M__–__1_ (*n* = 21) (Table 2). Plasmid sizes ranged between 100 and 137 kb. The dominant plasmid lineage was pST-3 (CC3), followed by pST-295 (CC2) and the novel pST-317 (CC3) (*ardA*_19, *pilL*_2, *repI1*_2, *sogS*_1, *trbA*_4) that was identified in a chicken isolate from Spain. IncI1-pST-3 plasmids revealed three different resistance gene patterns: *bla*_CTX__–__M__–__1_ and *sul2*, *bla*_CTX__–__M__–__1_ and *sul2* and *tet* (A), and *bla*_CTX__–__M__–__1_ and *sul2* and *aadA5* and *dfrA17*.

**TABLE 2:** Genomic location of ESBL/AmpC and MCR-1 genes and assignment to Inc groups.

**ESBL/AmpC/MCR-1**	**Location**		**Inc typing and pMLST**					**Plasmid size**
	ch (host)*	**pl**	**Inc type**	**ST**	**CC**	No. (host)*	No. (country)*	
CTX-M-1	0	23	IncI1α	3	3	16 (C), 3 (P)	10 (F), H(5), G (3), E (1)	100–137 kb
				295	2	1 (C)	1 (E)	~105 kb
				317	3	1 (C)	1 (E)	~100 kb
			IncN	1	n.d.	2 (P)	1 (F), 1 (G)	~43 kb
CTX-M-14	2 (P)	4	n.d.	n.d.	n.d.	4 (C)	4 (E)	110 kb
CTX-M-15	1 (Ca)	0	n.d.	n.d.	n.d.	1 (Ca)	1 (PL)	none
CTX-M-2	0	1	n.d.	n.d.	n.d.	1 (Ca)	1 (B)	n.d.
SHV-12	3 (C), 1 (P)	28	IncI1α	26	2	6 (C)	5 (E), 1 (F)	113–119 kb
				3	3	6 (C), 1 (P)	7 (E)	95–100 kb
				95	9	3 (C)	3 (NL)	115–116 kb
			IncX3	n.d.	n.d.	4 (C)	3 (H), 1 (E)	46 kb
			IncK2	n.d.	n.d.	1 (C)	1 (E)	~80 kb
			non-IncI1α	n.d.	n.d.	6 (C), 1 (P)	7 (E)	80–200 kb
SHV-2	0	1	non-IncI1α	n.d.	n.d.	1 (C)	1 (E)	~130 kb
TEM-52	0	5	IncI1α	36	3	2 (C), 1 (P)	2 (H), 1 (NL)	89.5 kb
			IncX1	n.d.	n.d.	1 (C)	1 (E)	38.7 kb
			non-IncI1α	n.d.	n.d.	1 (C)	1 (E)	~95 kb
CMY-2	0	22	IncI1α	12	10	9 (C), 1 (P)	6 (H), 2 (NL), 2 (E)	96–99 kb
				2	2	2 (C)	1 (E), 1 (H)	93–94 kb
			IncK2	n.d.	n.d.	7 (C)	4 (H), 2 (E), 1 (F)	80–90 kb
			IncA/C	3	n.d.	1 (Ca)	1 (B)	100 kb
			n.d.	n.d.	n.d.	2 (C)	1 (E), 1 (H)	90–95 kb
MCR-1	0	1	IncX4	n.d.	n.d.	1 (C)	1 (NL)	33.3 kb

*ch, chromosomal; pl, plasmid; C, chicken; P, pig; Ca, cattle; B, Belgium; G, Germany; E, Spain; F, France; H, Hungary; NL, Netherlands; PL, Poland; n.d., not determined.

Twenty of these plasmids showed the genomic backbone of pC60-108, a 108.6 kb plasmid determined in an *E. coli* strain from chicken in Switzerland in 2013 (Wang et al., 2014; Supplementary Document S3 and Supplementary Figure S3). Another two isolates from pigs in France and Germany revealed *bla*_CTX__–__M__–__1_ on IncN/pST-1 plasmids that were 42.4 kb in size and showed a similarity of 98.7% to IncN/pST-1 plasmid pL2-43 (Supplementary Document S3 and Supplementary Figure S4). Like pL2-43 (*E. coli*, lamb, Switzerland, 2013), our plasmids harbored macrolide resistance gene *mph*(A) in addition to *bla*_CTX__–__M__–__1_ (Wang et al., 2014).

In four avian *E. coli* isolates from Spain, we located the *bla*_CTX__–__M__–__14_ gene on a plasmid with unknown replicon type. The CTX-M-14 plasmids were about 110 kb in size and showed partial similarity with 55.1 kb CMY-2 plasmid 2016C-3936C1-unnamed2 (GenBank: CP018772) (Supplementary Document S3 and Supplementary Figure S5).

The only ESBL-producing strain identified among *E. coli* isolates from Poland (cattle, ST617, phylogroup C) was at the same time the only strain that harbored CTX-M-15. The *bla*_CTX__–__M__–__15_ gene was located on the chromosome and the genetic environment shows high similarity to that of human intra-abdominal infection ST4981 *E. coli* strain CH611_eco (CP017980.1) from China (data not shown). Although we could verify plasmid location of the *bla*_CTX__–__M__–__2_ gene of a bovine isolate from Belgium, a comparison with published plasmid sequences revealed no significant results.

#### SHV-12

The *bla*_SHV__–__12_ genes (*n* = 32) were predominantly located on IncI1α plasmids (50.0%), followed by IncX3 (12.5%), and IncK2 (3.1%) (Table 2). For seven isolates, we could not reconstruct plasmid sequences. According to results from sequence and S1-nuclease restriction analysis we termed them “non-IncI1”plasmids. Three isolates carried the *bla*_SHV__–__12_ gene on the chromosome. IncI1α plasmids (95.4–119 kb) were assigned to pST-26/CC2 (*n* = 6; Spain and France) pST-3/CC3 (*n* = 7; Spain), and pST-95/CC9 (*n* = 3; Netherlands). ST-26 and ST-95 plasmids co-harbored resistance genes *sul3*, *tet*(A), *cmlA*, *aadA1*, and *aadA2b*, whereas pST-3 plasmids only possessed the *bla*_SHV__–__12_ gene. Plasmid backbones of SHV-12 IncI1α plasmids revealed similarity to that of *bla*_SHV__–__12_-carrying plasmid pCAZ590 (GenBank: LT669764.1) that was identified in an *E. coli* isolate from chicken in Germany in 2011 (Supplementary Document S3 and Supplementary Figure S6).

The SHV-12 IncX3 plasmids were 46.3–46.4 kb in size and were highly similar (99.9%) to each other and to the reference plasmid pEC-244 GenBank: KX618704) (Supplementary Document S3 and Supplementary Figure S7). Like pEC-244 (*E. coli*, chicken feces, Netherlands), our plasmids harbored PMQR gene *qnrS1* in addition to *bla*_SHV__–__12_. The IncX3 plasmids also showed significant similarity to the plasmid backbone of the SHV-12 plasmid pUHKPC33 (*K. pneumoniae*, human patient, United States) (GenBank: NZ_CP011992).

#### TEM-52

Of five *bla*_TEM__–__52_ genes observed, three were encoded on a 89.5 kb IncI1α pST-36/CC-3 plasmid. These plasmids showed > 99% identity to reference plasmid pESBL-117 (urine, human patient, the Netherlands) (GenBank: CP008734.1) (Supplementary Document S3 and Supplementary Figure S8) and carried no additional resistance genes. One chicken isolate from Spain harbored the *bla*_TEM__–__52_ gene on a 38.7 kb IncX1 plasmid with 99.7% identity to reference plasmid pDKX-TEM-52 (JQ269336.1) that was isolated from chicken meat in Denmark in 2006 (Johnson et al., 2012).

#### CMY-2

The *bla*_CMY__–__2_ gene that was present on plasmids with replicon types IncI1α (*n* = 12), IncK2 (*n* = 7), and IncA/C (*n* = 1) (Table 2). The 95–122 kb CMY-2 IncI α plasmids were classified into pMLST types pST-12 (*n* = 10) and pST-2 (*n* = 2), and carried not additional resistance gene.

Nine pST-12 plasmids revealed high similarity to the 99 kb-plasmid p11-004736-1-7_99 (*S.* Heidelberg, cattle, Canada, 2011) (Supplementary Document S3 and Supplementary Figure S9; Labbe et al., 2016). IncI1α/pST-2 plasmids from chickens in Spain and Hungary showed > 99% similarity to plasmid pSA01AB09084001_92 (*S*. Heidelberg, chicken, cecal content, Canada, 2009) (Supplementary Document S3 and Supplementary Figure S10; Labbe et al., 2016). The backbone of IncK2-CMY-2 plasmids (83–86 kb) was highly similar to the *bla*_CMY__–__2_-carrying plasmid pDV45 (85.9 kb) from poultry retail meat (Supplementary Document S3 and Supplementary Figure S11; Seiffert et al., 2017). While CMY-2 plasmids of the IncI1α and IncK2 replicon types encoded no further resistance genes, the IncA/C-pST-3 plasmid (99.9 kb) observed in a bovine *E. coli* isolate from Belgium carried resistance genes *dfrA12*, *tet*(A), *aadA2*, *aph(3″)-Ib*, *aph(6)-Id*, *floR*, *sul1*, and *sul2* in addition. It revealed a plasmid backbone of the 135.2 kb plasmid pSH163_135 (*S.* Heidelberg, turkey, United States, 2002) (Supplementary Document S3 and Supplementary Figure S12; Han et al., 2012). It also showed high similarity to the 96 kb *K. pneumoniae* plasmid pKP_Goe_024-2, which was isolated from the abdominal drainage fluid from a human patient in Germany in 2014 and carried a *bla*_*OXA*__–__48_ instead of a *bla*_CMY__–__2_ gene. Two avian isolates from Spain and Hungary carrying CMY-2 plasmids without known replicon types revealed a genomic backbone of 55.1 kb-plasmid 2016C-3936C1 unnamed2 (*E. coli* O157, human, United States) (GenBank: CP018772) (Supplementary Document S3 and Supplementary Figure S13).

#### MCR-1

The *mcr-1* gene in avian *E. coli* isolate IHIT31981 was encoded on a 33.3 kb IncX4 plasmid that was almost identical (99.9% nucleotide sequence identity) with other globally distributed MCR-1 IncX4 plasmids, such as pMCR-1-CT (GenBank: CP018773.2), determined in an *E. coli* isolate from a human patient in the United States.

### Phylogenetic Grouping

**FIGURE 2:**
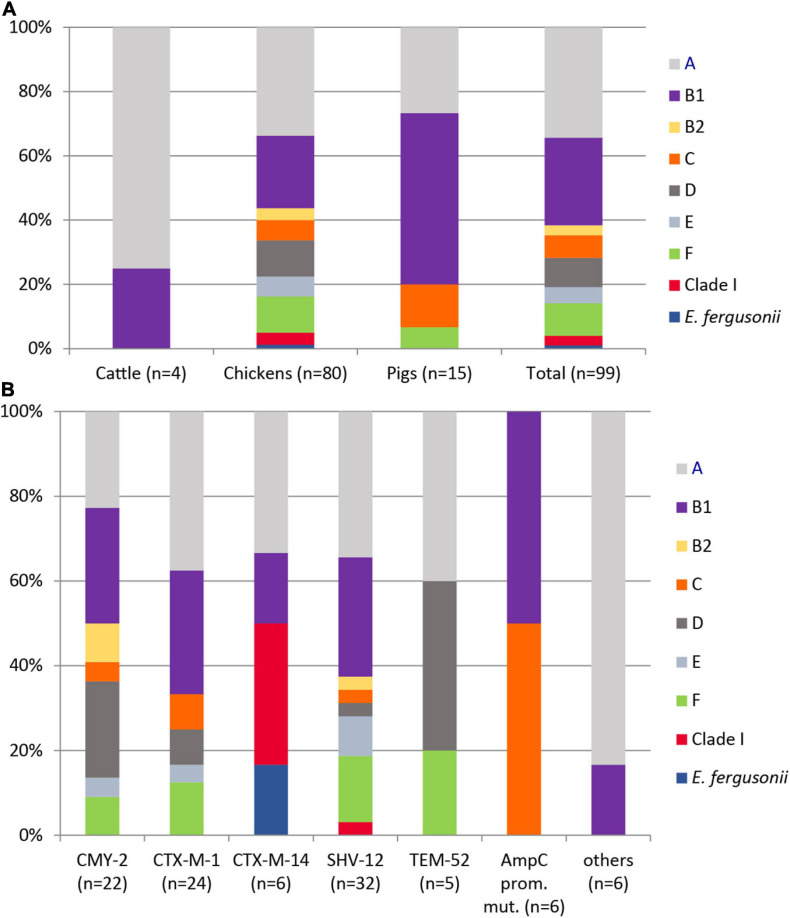
Distribution of phylogenetic groups among 99 ESC-non-S/ESC-R *Escherichia* spp. isolates with respect to animal host **(A)** and ESBL/pAmpC genes and AmpC-promoter mutations **(B)**.

Using the ClermonTyping method and its associated web-interface ClermonTyper, 98 isolates were verified as *E. coli* and one, as expected, as *E. fergusonii.* The majority of the 98 *E. coli* isolates were assigned to phylogenetic groups A (34.7%) and B1 (27.6%). Group C, a newly defined phylogroup within the formerly highly diverse group A (Clermont et al., 2013), was identified in 7.1% of the isolates. Phylogenetic groups B2 (3.1%), D (9.2%), and F (10.2%), the latter one representing a novel group D-related cluster, were less prevalent. Three isolates (3.0%) from chickens in Spain belonged to clade I. Five isolates (5.1%) were assigned to phylogenetic group E (Figure 2A). All phylogroups B2 and D and 90% of group F isolates, often referred to as extraintestinal pathogenic *E. coli* (ExPEC) groups, were from chickens. Those phylogroups regarded as commensals (A, B1, C) were detected in 62.5% of chicken, 100% of cattle, and in 93.3% of pig isolates.

The distribution of ESBL/pAmpC types revealed no significant association to the phylogenetic group of a strain (Figure 2B). Among 12 antibiotic classes tested, phylogroup B1 strains showed the highest mean number of resistance (resistant to 5.19 ± 1.36 classes), followed by isolates belonging to group E (4.8 ± 0.87), A (4.79 ± 1.28), clade I (4.67 ± 1.33), F (4.4 ± 1.05), and D (4.11 ± 1.04). Group C (3.71 ± 0.89) and B2 (3.67 ± 0.33) isolates revealed the lowest antimicrobial resistance with regard to different antibiotic classes.

### Distribution of Serotypes

Overall, 48 different serotypes were identified among the 99 ESC-non-S/ESC-R isolates (Supplementary Document S2). Fifteen isolates, including *E. fergusonii*, were O not typable. Twenty-nine serotypes appeared as singletons. Twenty-one isolates revealed serotypes that are frequently associated with ExPEC including O1, O2, and O6 (*n* = 2 each), O7 (*n* = 6), O8 (*n* = 8), and O78 (*n* = 1) (Ewers et al., 2007). In addition, the CMY-2-ST131 isolates revealed serotype O25b:H4, which is highly linked with this ST. Moreover, one CMY-2 producing isolate (IHIT32055, ST533) from a chicken in Hungary revealed serotype O157:H10, which is frequently observed among enterohemorrhagic strains. Other serotypes that are commonly recorded from EHEC and EPEC (O26, O103, O111, and O145) or from ETEC (O45, O138, O139, O141, O147, and O149) were only rarely detected (two TEM-51 O149:H20 isolates from chickens in Hungary; two avian O45 isolates from Spain (CMY-2) and France (CTX-M-1).

### Distribution of Virulence-Associated Genes (VAGs) and Pathotyping

We tested our 99 *Escherichia* spp. strains for 800 VAGs associated with intestinal and extraintestinal pathogenic *E. coli* pathovars. More than 300 of these VAGs, belonging to the categories adhesion (*n* = 98), iron acquisition (*n* = 45), invasion and protection (*n* = 42), secretion system (*n* = 115), and autotransporter/toxin (*n* = 19) were identified in the isolates with different frequencies (Supplementary Document S1).

#### Mean Number of VAGs

The mean number (± SD) of VAGs detected was 113.04 ± 15.84. The VAG content was highest in phylogroup D isolates (134.4 ± 13.7), followed by groups E (130.8 ± 14.1), C (127.1 ± 14.9), B2 (119. 7 ± 2.7), F (115.6 ± 12.1), B1 (113.7 ± 13.3), clade I (113.0 ± 2.0), and group A (102.6 ± 14.0). Mean VAG numbers according to ESBL/pAmpC genes carried by the isolates were in decreasing order: 125.2 ± 11.6 (AmpC-promoter mutation); 119.5 ± 16.7 (CMY-2); 114.5 ± 22.0 (CTX-M-1); 113.0 (SHV-2, one isolate); 113.0 ± 6.0 (TEM-52); 111.4 ± 10.1 (SHV-12); 104.0 (CTX-M-15, one isolate); 99.2 ± 16.7 (CTX-M-14); 84.0 (CTX-M-2, one isolate) (Supplementary Table S4). Isolates from pigs (117.0 ± 17.5) and chickens (113.5 ± 14.9) harbored significantly higher numbers of VAGs than isolates from cattle (89.5 ± 9.6).

#### InPEC-Related VAGs

The majority of isolates carried none of the 43 InPEC-related VAGs (Table 3). One or two of these VAGs were present in 43.4% of the isolates, and only 6.1% of the isolates carried 8–12 genes. None of the isolates possessed invasion plasmid antigen gene *ipaH*, which would indicate the presence of enteroinvasive *E. coli* (EIEC). Most typical factors associated with the enteroaggregative *E. coli* (EAEC) pathotype, such as aggregative adherence fimbriae AAF/I to AAF/V (*aggA*, *aafABCD, agg3A*, *agg4A*, *agg5A*), cytotoxic autotransporter protease Pet (*pet*), antiaggregation protein dispersin (*aap*, *aatPABDC*), or a type IV secretion system (*aai*), as well as the global regulator of these genes, AggR (*aggR*, *agg* variants), were also not present. Only three factors that are commonly, though not exclusively, present in EAEC, namely enteroaggregative heat-stable enterotoxin EAST-1 (*EAST-1*, 31.3%), serine protease/mucinase Pic (*pic*, 6.1%) and the enteroaggregative immunoglobulin repeat protein Air (*air*, 21.2%), were detected in our isolates. Due to the absence of genes for cytotoxic necrotizing factors (*cnf1-cnf3*) and cytolethal distending toxins (*cdtA-cdtC*) and of shiga toxin genes (*stx1* and *stx2* variants) the presence of NTEC (necrotoxigenic *E. coli*) and STEC (Shigatoxin-producing *E. coli*) was excluded (Supplementary Document S1).

**TABLE 3:** Distribution of InPEC- and ExPEC-related virulence-associated genes (VAGs) in ESC-non-S/ESC-R *E. coli* isolates.

**Pathotype**	**Positive isolates**	
	**n**	**%**
**InPEC^*a*^**		
0 genes	15	15.2
1–2 genes	43	43.4
3–4 genes	16	16.2
5–7 genes	0	0
8–12 genes	6	6.1
EAEC, EIEC, ETEC, NTEC	0	0
EPEC (typical/atypical)	0/6	0/6.1
STEC (including EHEC, EDEC)	0	0
**ExPEC**		
**Group 1 (ExPEC)^*b*^**		
0 genes	75	75.8
1 gene	22	22.2
**≥2 genes**	**2**	2.0
**Group 2 (UPEC)^*c*^**		
ExPEC genes and *cnf1/cnf2* and *hlyD*	0	0
Group 3 (APEC)^*d*^		
0 genes	18	18.2
1–2 genes	30	30.3
**≥3 genes**	**51**	**51.5**
**Group 4 (ExPEC-like)^*e*^**		
3–5 genes	9	9.1
6–10 genes	31	31.3
11–15 genes	35	35.4
16–20 genes	17	17.2
21–26 genes	7	7.1

*^*a*^Isolates carrying one or more of 43 InPEC-related VAGs among the categories (i) toxin: cnf1, cnf2, cnf3, cdtA, cdtB, cdtC, EAST-1, eltA, eltB, esta, estb, stx1, stx2; (ii) adhesion: aaf, aggA, aggR, aidA, bfpA, eae, f17, fae, fan, fas, fed, iha, stgA, tir; (iii) invasion, secrection, autotransporter: ces, eatA, escN, espD, etgA, ipaH, lifA, pet, pic, sepDL, tia, tibA; (iv) others: aap, air, ler, nleA (see Supplementary Document S1 for detailed distribution). ^*b*^Isolates with genes (afa/dra, focG, iutA, kpsMTII, papA and/or papC, sfa/focDE) that have been suggested to define a strain as an ExPEC; ≥ 2 genes = ExPEC, according to Johnson et al. (2003). ^*c*^Isolates with ≥ 2 genes plus cnf1/cnf2 and hlyD, which are more specifically related to uropathogenic E. coli (UPEC). ^*d*^Isolates with genes (iroN, iss, iutA, ompT, hlyF) that have been suggested to define avian ExPEC; ≥ 3 genes = avian pathogenic E. coli (APEC) according to Johnson et al. (2008). ^*e*^Isolates with genes (n = 32) or gene clusters (n = 17) frequently, but not exclusively associated with ExPEC; isolates with > 10 genes/gene clusters = ExPEC-like (see Supplementary Table S3 for list of VAGs).*

None of our isolates carried ETEC-related toxin genes (*esta, estb, eltA, eltB*). Few isolates harbored genes for ETEC-related fimbriae F4 (*fae*, 4.0%, 2 × chicken, 1 × cattle, 1 × pig), F5 (*fan*, 3.0%, 3 × chicken), and F17 (f17, 1.0%, 1 × cattle), while F6 and F18 fimbriae genes were not detected. Other VAGs often reported for ETEC were found in 31.0% (*EAST-1*—heat-stable enterotoxin 1 gene), 30.3% (*aidA*–AIDA-I-like adhesion gene), 5.1% (*iha*–bifunctional enterobactin receptor adhesin protein gene), and 4.0% (*tia*—toxigenic invasion locus gene) of the isolates.

Six isolates (6.1%) represented atypical enteropathogenic *E. coli* (aEPEC) as they harbored intimin gene *eae* and lacked bundle-forming pili adhesin genes *bfpA-L*, which together with *eae* are indicative for typical EPEC (Table 3). Intimin genes revealed ≥ 99.8% nucleotide sequence identity to *eae* type β1 (GenBank AF200363.1). All strains additionally harbored the *tir* (translocated intimin receptor) gene, and most isolates additionally possessed several genes encoding for type III secretion proteins, effectors, and regulators (*esc, ces, epa, epr, etgA, mpc, sepDL*), for locus of enterocyte and effacement (LEE) regulator gene *ler*, and for non-LEE-encoded type III effector genes (*nle*). The ST10 chicken isolate IHIT32040 was the only aEPEC strain that carried genes for the AIDA-I-like adhesin protein (*aidA*) and for Stg fimbriae (*stgABCD*). Three of the aEPEC strains additionaly harbored the porcine attaching and effacing-associated gene *paa* that promotes adherence to intestinal epithelial cells in a characteristic A/E pattern (Batisson et al., 2003).

#### ExPEC-Related Genes

More than half of the 99 isolates fulfilled the criteria to be classified as avian ExPEC, also termed avian pathogenic *E. coli* (APEC). In detail, 51.5% of our isolates harbored at least three of the five VAGs *iroN, iss, iutA, ompT*, and *hlyF* that have been suggested to define APEC (Table 3; Johnson et al., 2008). A few years earlier, Johnson and Russo (2005) defined the group of ExPEC on the basis of the presence of ≥ 2 VAGs or gene combinations, including genes for P fimbrial genes (*papAH* and/or *papC*), S and FIC fimbriae (*sfa/focDE*), Dr. antigen-binding adhesin (*afa/draBC*), group II capsule polysaccharides (*kpsMTII*), and ferric aerobactin receptor (*iutA*) (Johnson and Russo, 2005). This definition includes all pathotypes that were primarily “ill-defined,” including uropathogenic *E. coli* (UPEC), neonatal meningitis-associated *E. coli* (NMEC), sepsis-causing *E. coli* (SEPEC), and APEC. Following this definition, only two of our isolates qualified as ExPEC, namely a CMY-2-positive avian isolate from Spain (phylogroup D, ST38) and a CTX-M-1-producing porcine isolate from Germany (phylogroup B1, ST453).

As current evidence suggests that no single virulence determinant renders an ExPEC isolate capable of causing site-specific disease, we additionally screened our isolates for an extensive set of 49 VAGs (Supplementary Table S3) that have previously been linked with different ExPEC pathovars in order to categorize them as ExPEC-like pathovar (Dale and Woodford, 2015). Seven isolates, all from chicken, possessed 21–26 ExPEC-related VAGs and belonged to phylogroups F (*n* = 4), B2 (*n* = 3), and C (*n* = 1). Another 17 isolates, including A, B1, C, and D strains, harbored 16–20 genes. The majority of strains harbored 6–10 (31.3%) or 11–15 (35.4%) of the VAGs tested. All strains were negative for afimbrial adhesin Afa (*afaA-G*), Dr. fimbriae (*draA-E, draP*) and S-fimbriae (*sfaA-H*, *sfaS*), as well as for toxin genes *cnf1-3* and *sat*, i.e., for virulence determinants that are frequently observed among UPEC. Invasion-related gene *gimB*, which has been reported for APEC and NMEC, was also not detected. Genes detected only once were α-hemolysin gene *hlyA and hlyD*, salmochelin receptor gene *iutA*, and Expec adhesin gene *yqi*. Other ExPEC-related genes, encoding P fimbriae (*pap*; 4.0%), K1 capsule synthesis (*neu*; 4.0%), uropathogenic specific factor (*usp*; 8.1%), serine protease Pic (*pic*; 6.1%), vacuolating autotransporter toxin Vat (*vat*; 3.0%), adherence protein Iha (*iha*; 5.1%), invasion factor IbeA (*ibeA*; 6.1%), or antigen 43 (*agn43*; 6.1%) occurred in 2–8 of the isolates. Between 91.0 and 99.0% of the isolated possessed Curli fiber (*csg*) and type I fimbriae (*fim*) genes. The most often detected iron acquisition genes were *ent* (98.0%), *sit* (66.7%), *iuc* (54.5%), and *iro* (46.5%), coding for enterobactin siderophore, iron transport system, aerobactin siderophore, and a salmochelin siderophore system, respectively. Among the protectins, increased serum survival protein *iss* (77.8%) and outer membrane exclusion protein *traT* (75.8%) were most prevalent (Supplementary Table S3).

### Clonal Diversity

With 51 known and two novel types, we found a wide variety of multilocus sequence types (STs) among our strains. For isolates from broilers (*n* = 80), pigs (*n* = 15) and cattle (*n* = 4) we determined 42, 14, and 3 different STs, respectively (Table 4). Thirty-two of these STs have been assigned to 18 clonal complexes (CCs), while 21 STs did not cluster within one of the CCs defined in the Enterobase *E. coli* database (Supplementary Document S2)^7^. The most often identified STs were ST117 and ST10 (*n* = 6 each), ST665 (*n* = 5) ST23, ST38, ST155 (*n* = 4 each), ST101, ST354, ST4980, ST752, ST770, and ST88 (*n* = 3 each). ST648 and ST131, two globally distributed lineages that are frequently associated with ESBL/AmpC production were identified in a CTX-M-1-producing chicken isolate from Spain (ST648) and in two CMY-2 positive chicken ST131 isolates from different farms in Hungary. A correlation between STs and animal species or country of origin was not evident. In addition, ESBL and AmpC β-lactamase types, as well as plasmid replicon types showed a random distribution among the different STs.

**TABLE 4:** Multilocus sequence types and ESBL/AmpC types of 99 ESC-non-S/ESC-R *Escherichia* spp. from livestock animals.

**Chicken**		**Chicken (continued)**	
**ST (n)**	ESBL/pAmpC*	**ST (n)**	**ESBL/pAmpC**
ST10 (5)	SHV-12 (3), CTX-M-1, CTX-M-14	ST1594 (2)	CTX-M-1 (1)
ST23 (4)	CTX-M-1, CMY-2, none (2)	ST1621 (1)	SHV-12
ST38 (4)	CMY-2 (3), CTX-M-1 (2)	ST1730 (1)	none
ST48 (2)	SHV-12 (2)	ST4980 (3)	TEM-52 (1), SHV-12, CTX-M-1
ST68 (1)	CMY-2	ST3406 (1)	CTX-M-1
ST88 (1)	CTX-M-1	ST3994 (1)	none
ST93 (1)	CMY-2	ST4118 (2)	TEM-52 (2)
ST101 (2)	SHV-12, CMY-2	ST4243 (1)	CMY-2
ST117 (6)	CTX-M-1 (2), CMY-2, TEM-52,	ST7104 (1)	CTX-M-1
	SHV-12/CMY-2	ST7852** (1)	CTX-M-14
ST131 (2)	CMY-2 (2)	ST10807 (1)	CTX-M-1
ST135 (1)	SHV-12		
ST154 (1)	SHV-12	**Swine**	
ST155 (4)	SHV-12 (3), CTX-M-1	**ST (n)**	**ESBL/pAmpC**
ST156 (2)	none (2)	ST10 (1)	CTX-M-1
ST162 (1)	CTX-M-1	ST58 (1)	CTX-M-1
ST189 (1)	TEM-52	ST56 (1)	CTX-M-1
ST354 (3)	SHV-12 (3)	ST88 (2)	SHV-12 (1), none (1)
ST371 (1)	CMY-2	ST101 (1)	SHV-12, TEM-52
ST398 (2)	SHV-12 (2)	ST345 (1)	SHV-12
ST453 (1)	CMY-2	ST398 (1)	CTX-M-1
ST533 (1)	CMY-2	ST453 (1)	CTX-M-1
ST648 (1)	CTX-M-1	ST847 (1)	none
ST665 (5)	CMY-2 (2), SHV-2, SHV-12,	ST1147 (1)	CTX-M-14
	CTX-M-1	ST1304 (1)	CMY-2
ST752 (3)	SHV-12 (2), CMY-2	ST2197 (1)	CTX-M-14
ST770 (3)	CTX-M-14 (2), SHV-12	ST10813 (1)	none
ST997 (2)	SHV-12 (2)		
ST1137 (1)	CTX-M-1	**Cattle**	
ST1158 (1)	SHV-12	**ST**	**ESBL/pAmpC**
ST1246 (1)	SHV-12	ST515 (1)	CMY-2
ST1304 (1)	CMY-2	ST617 (1)	CTX-M-15
ST1431 (1)	SHV-12	ST744 (2)	CTX-M-1, CTX-M-2
ST1551 (1)	CTX-M-1		

*In case an ESBL/AmpC type appears more than once, the number is indicated in brackets.**Escherichia fergusonii (IHIT32041).

**FIGURE 3:**
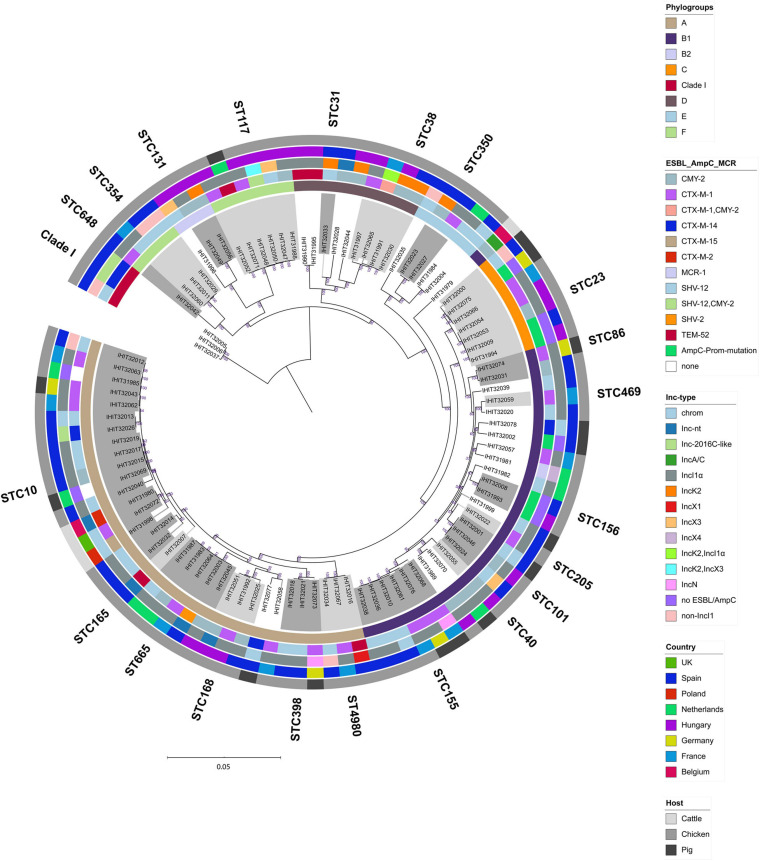
Maximum-likelihood tree of 98 *E. coli* genomes (*E. fergusonii* isolate IHIT32041 was not included). The phylogeny is based on 1336 orthologous genes and was calculated by RAxML. Graphical visualization was achieved by iTOL v5. Colored rings represent (from inner to outer ring the (i) phylogroup, (ii) ESC-non-S/ESC-R resistance determinant, (iii) ESBL/AmpC/MCR-1 plasmid replicon type, (iv) country of isolation, and (v) host.

We further determined the relatedness of strains from different animal species and countries by WGS comparison. Although the application of comparative core genome analysis of 98 *E. coli* isolates, based on 1,330 orthologous genes, achieved a much higher resolution regarding the genetic relatedness of the ESBL/pAmpC/MCR-1 strains (SNP-based comparison of 1,330 genes vs. allele-based comparison of seven MLST genes), it basically reflected the high genetic diversity observed by MLST analysis (Figure 3). As expected, isolates forming one ST and/or CC by based on MLST data as well as isolates that shared the same phylogenetic groups almost always clustered together in the cgMLST scheme. In contrast, neither ESBL/AmpC-types nor plasmid Inc types followed the phylogenetic distribution of the isolates but were broadly distributed among the different cgMLST clusters.

### Correlation of Antimicrobial Resistance and VAG Pattern With Metadata of the Isolates

We first visualized the clustering pattern, i.e., AMR genes categorized according to their mode of action with the metadata of isolates (Supplementary Figure S1). As expected, there was an association between the category “antibiotic inactivation” and ESBL/pAmpC types (*p* < 0.001). In addition, single AMR genes showed associations with either host (e.g., *floR*, *aph(3)-Ia*, *aph(3)-Ib*, *aph(6)-Id*, and *mphA* to cattle; *aph(3)-Ib*, *aph(6)-Id, dfrA12*, and *pmrE* to chicken), country, ESBL/AmpC-type, and phylogenetic group.

The same analysis was done using VAGs grouped into different categories as the clustering pattern (Supplementary Figure S2). Significant associations were predominantly observed between VAGs and the phylogenetic group. In total 36 adhesion-related, 11 autotransporter and toxin genes, 22 invasion and protectin genes, 14 iron acquisition genes and 33 secretion systems genes revealed several *P*-values < 0.001. In contrast, only 14, 21, and 7 VAGs showed significant associations with the variables host, country, and ESBL/AmpC type.

## Discussion

This study focused on the identification and investigation of ESBL and pAmpC genes in ESC-non-susceptible/ESC-resistant commensal *E. coli* isolates. Special attention was paid to the antimicrobial resistance phenotype of the isolates and their plasmids with regard to replicon type and genetic plasmid structure. We further investigated the molecular epidemiology of *E. coli* isolates with respect to their genome-based phylogeny and assignment to intestinal and extraintestinal *E. coli* pathotypes. The presence of potentially virulent and antimicrobial resistant ESBL/pAmpC-producing *E. coli* originating from healthy production animals could be a significant issue for human and animal health.

One hundred ESC-non-susceptible/ESC-resistant isolates were identified among 2,993 commensal *E. coli* isolates collected over a time period of 25 months (February 2013–March 2015) from different livestock species and European countries. The highest rate of ESC-non-susceptible/ESC-resistant isolates was detected in poultry (8.0%), whereas pigs (1.3%) and cattle (0.5%) revealed comparable low numbers of such isolates. These findings are fairly in agreement with the numbers reported by the European Food Safety Authority (EFSA) and the European Centre for Disease Prevention and Control (ECDC). Here the number of presumptive ESBL and AmpC phenotypes identified in indicator *E. coli* isolates rated to 5.4% for broiler flocks in 2014 [EFSA (European Food Safety Authority) and ECDC (European Centre for Disease Prevention and Control), 2016], and to 1.5 and 2.0% for fattening pigs and calves under 1 year of age, respectively, in 2015 [EFSA (European Food Safety Authority) and ECDC (European Centre for Disease Prevention and Control), 2017]. The lower number observed for cattle in our study might be due to the mixed origin of isolates according to animal age. Different studies could show that veal calves harbor higher proportions of ESBL/AmpC-producing bacteria than beef cattle [Haenni et al., 2014; EFSA (European Food Safety Authority) and ECDC (European Centre for Disease Prevention and Control), 2017]. Recent data from 2017 to 2018 indicated that the occurrence of presumptive ESBL- and/or AmpC-producing indicator *E. coli* isolates was still generally low in reporting European countries, ranging from 0.6 to 6.3% in isolates from pigs, from 1.2 to 5.3% in isolates from calves and from 0.6 to 7.9% in isolates from broilers [EFSA (European Food Safety Authority) and ECDC (European Centre for Disease Prevention and Control), 2020]. Carbapenemase producing (CP) *E. coli* were not detected in our study, which may be due to the absence of such isolates and/or the cultivation of the samples on non-selective media. The presence of CP bacteria on livestock farms was first reported in 2012 in Germany (Fischer et al., 2013). Since then, increasingly emerged in different livestock species and countries (Anderson and Boerlin, 2020). Despite of these findings, the overall prevalence of CPs in livestock is still low as shown by the results from the specific carbapenemase producers monitoring in European countries between 2016 and 2018 [EFSA (European Food Safety Authority) and ECDC (European Centre for Disease Prevention and Control), 2020].

Almost all isolates (97.0%) from our study revealed an MDR phenotype, i.e., resistance to antimicrobial agents of at least three classes, and 29.0% of the isolates showed resistance to ≥ six classes of antimicrobial agents. This is consistent with previous studies from different countries. Ninety percent of cefotaxime and/or ciprofloxacin resistant, commensal *E. coli* isolated from food-producing animals in Belgium were resistant to several other antibiotics (Lambrecht et al., 2018). MDR was also observed in 88.1% of ESBL-producing isolates obtained between 2008 and 2014 from diseased food-producing animals in Germany (Michael et al., 2017). In this study, 39.4% of the isolates, predominantly from cattle, were resistant to six antibiotic classes. Several authors assumed a correlation between antimicrobial usage in food-producing animals and the prevalence of AMR bacteria in those animals. According to publicly available national or international reports from seven European countries, the level of veterinary use of specific antimicrobials strongly correlates to the level of resistance toward these agents in commensal *E. coli* isolates in pigs, poultry and cattle (Chantziaras et al., 2014). A significant correlation between antimicrobial use and resistance in commensal *E. coli* was also demonstrated for livestock animals in Belgium (Callens et al., 2018). In our study, isolates were frequently (32–100%) resistant to ampicillin, sulfisoxazole, tetracycline, trimethoprim/sulfamethoxazole, and ciprofloxacin. Antimicrobial agents of such classes are among the most sold agents to veterinarians in different European countries (Lekagul et al., 2018; Roth et al., 2019), indicating that AMR and frequent antibiotic usage could coincide to an extent that remains to be determined.

The identification of eight different ESBL/pAmpC types indicated a high diversity of β-lactamases among our isolates, which is consistent with previous studies (Valentin et al., 2014; Ceccarelli et al., 2019). The two major ESBL genes were *bla*_SHV__–__12_ (35.6%) and *bla*_CTX__–__M__–__1_ (24.2%), while *bla*_CMY__–__2_ (22.2%) was the only pAmpC gene detected. The CTX-M-1 β-lactamase is the most common ESBL in livestock animals and food, and also SHV-12 ranks among the most predominant ESBL types within Enterobacteriaceae of diverse origins (Ewers et al., 2012; Valentin et al., 2014; Alonso et al., 2017; Liakopoulos et al., 2018). Regarding *bla*_CMY__–__2_, an increasing number of *E. coli* carrying this gene was reported in the European livestock production in recent years (Ewers et al., 2012; Larsen et al., 2012; Irrgang et al., 2018; Liakopoulos et al., 2018; Pietsch et al., 2018; Ceccarelli et al., 2019; EFSA (European Food Safety Authority) and ECDC (European Centre for Disease Prevention and Control), 2019). Consistent with our findings (25.0% of ESC-R/ESC-non-resistant poultry isolates carried *bla*_CMY__–__2_), a prevalence of more than 30% among global ESC-R *E. coli* expressed CMY-2, particularly in poultry (Ewers et al., 2012).

ESBL genes *bla*_CTX__–__M__–__14_ (6.1%, chicken and pig) and *bla*_TEM__–__52_ (5.1%, chicken and pig) were present in lower numbers among our isolates, while *bla*_SHV__–__2_ (chicken), *bla*_CTX__–__M__–__2_ (cattle) and *bla*_CTX__–__M__–__15_ (cattle) occurred only once. Together with CTX-M-1 and CTX-M-15, CTX-M-14 represents one of the most common ESBL type in *E. coli* isolates from cattle and its largely moderate occurrence in *E. coli* isolates derived from chickens and pigs has also been demonstrated (Ewers et al., 2012; Palmeira et al., 2020).

AmpC activity due to chromosomal *ampC* promoter mutations was found in 6.1% (6/99) of our isolates. All seven isolates revealed a mutation pattern previously described as *ampC* type 03 (Mulvey et al., 2005). This type was reported as one of the most prevalent variants among cefoxitin-resistant clinical *E. coli* isolates in different studies (Mulvey et al., 2005; Peter-Getzlaff et al., 2011). The *ampC* promoter alterations created an alternate displaced promoter whose mutation at position −42 (C > T) is thought to have strong effect on promoter strength and consequently on hyperproduction of *ampC* (Caroff et al., 2000).

The majority of the 70 ESBL and 22 pAmpC genes determined in this study were plasmid located (84/92; 91.3%). IncI1α was the most represented plasmid family (52/92; 56.5%) that occurred in 57.5% (46/80) of chicken and in 40.0% (6/15) of pig isolates, followed by IncK2 (8/92; 8.7%; all from chicken), IncX3 (4/92; 4.3%, chicken), IncN (2/92; 2.2%, pigs), IncA/C (1/92; 1.2%, cattle), and IncX1 (1/92; 1.2%, chicken). For 17.4% (16/92) of our ESBL/pAmpC plasmids, we could not assign a definite Inc type. Previous studies highlighted the challenge in reconstructing plasmids encoding resistance genes from WGS, mostly due to the lack of long reads (Orlek et al., 2017; Arredondo-Alonso et al., 2018; Ludden et al., 2019).

CTX-M-1-producing isolates belonged largely (75.0%) to IncI1α/pST-3 plasmids, which represent a globally distributed, genetically highly similar plasmid lineage that is disseminated in livestock animals and humans in Europe (Carattoli, 2008; Zurfluh et al., 2014; Irrgang et al., 2018; Touzain et al., 2018).^8^ IncI1 plasmids are highly conjugative, and transmission from commensal to pathogenic bacteria poses a risk for animal and human health (Carattoli, 2013). One of our CTX-M-1 plasmids was assigned as pST-295, a plasmid type that was first identified in a commensal CTX-M-1 ST1604 *E. coli* isolate from chicken in Denmark in 2015 (Liu et al., 2019). In that study, four novel CTX-M-1 IncIα plasmid lineages (pST-293–pST-296) were determined among livestock isolates. Together with our finding of novel type pST-317, this could reflect a possible diversifying evolutionary process in these otherwise highly conserved plasmids. Noteworthy, the plasmid transfer of several plasmid types, including IncI1-pST3-CTX-M-1 and IncI1-pST295-CTX-M-1 could be significantly increased *in vitro* in a strain-dependent manner following exposure to the antibiotics cefotaxime, ampicillin, and ciprofloxacin (Liu et al., 2019).

The diverse population of *E. coli* isolates carrying CTX-M-1 IncI1 plasmids (21 IncI1 plasmids among 18 different MLST types) suggests that the dissemination of this ESBL gene is not due to the spread of single clonal lineages but is more likely the result of vertical transmission of plasmids by horizontal transfer. Also Irrgang and co-workers (2018) reported 51 different STs among 89 CTX-M-1 producing *E. coli* isolates from German food samples.

In agreement with recent reports, SHV-12 was mainly encoded on IncI1α plasmids (50%) in our isolates, followed by IncX3 (12.5%) and IncK2 (3.1%) (Huijbers et al., 2014; Alonso et al., 2017; Apostolakos et al., 2020). When typing 23 SHV-12 positive *E. coli* from human, animal and food sources, Alonso et al. (2017) identified IncI1 as predominant replicon type (73.9%), while IncK2 (13.0%) and IncX3 (4.3%) were less prevalent (Alonso et al., 2017). IncI1-pST-3-SHV-12 was suggested as a poultry-associated plasmid lineage, whereas IncI1-pST26-SHV-12 plasmids were associated with a wide host range contributing to the spread of *bla*_SHV__–__12_ genes among different environments (Accogli et al., 2013; Alonso et al., 2017). We cannot conclude such an association from our data, as all pST-26 plasmids were from chicken isolates and pST-3 plasmids from chicken and pig isolates. However, this lack of correlation may be due to the predominance of chicken isolates among our collection.

Our SHV-12 IncX3 plasmids were almost identical in plasmid backbone and *bla*_SHV__–__12_ flanking region to pEC-244, which was the first completely sequenced IncX3 plasmid of animal origin. In this study, IncX3 plasmids from humans and animals exhibited remarkable synteny in their backbone and differed only in their *bla*_SHV__–__12_-flanking region (Liakopoulos et al., 2018). IncX3 plasmids are conjugative and highly stable, while they exert no fitness cost on their bacterial host, thus highlighting the epidemic potential of these plasmids.

In eight *E. coli* isolates from chickens, *bla*_CMY__–__2_ (*n* = 7) and *bla*_SHV__–__12_ (*n* = 1) has been linked to plasmids of the recently defined incompatibility group IncK2 (Seiffert et al., 2017). IncK2 plasmids are mainly associated with the spread of *bla*_CMY__–__2_ and *bla*_CTX__–__M__–__14_ genes in Europe, particularly in Spain and the United Kingdom, and are frequently reported from *E. coli* from livestock sources (Dhanji et al., 2012; Rozwandowicz et al., 2018; Apostolakos et al., 2020). de Been et al. (2014) identified *bla*_CMY__–__2_ carrying IncK2 plasmids in human and poultry isolates belonging to evolutionary distinct backgrounds, suggesting that these plasmids efficiently spread through *E. coli* populations of different reservoirs. We also identified IncK2 plasmids in isolates belonging to different phylogenetic groups (B2, D, E, and F) and to six different MLST types, including ST38, ST117, and ST131. The most frequent replicon type identified among *bla*_CMY__–__2_ carrying plasmids in our study was IncIα (*n* = 12 isolates from Netherlands, Hungary, and Spain). IncI1α and IncK2 were also found as major replicon types of *bla*_CMY__–__2_ carrying plasmids from *E. coli* isolates from humans, animals and food in Germany (Pietsch et al., 2018). Due to high sequence identity of plasmids, also shown for several plasmids in our study, the authors suggested that plasmid-mediated, rather than clonal spread likely plays an important role for emergence and transmission of *bla*_CMY__–__2_ between aminals and humans.

Some of the clones (ST10/A, ST23/C, ST38/D, ST117/F, and ST155/B1) detected in our study more than once have been previously associated with ESBL/pAmpC phenotypes and are widely spread among different environments, including clinical settings (Ewers et al., 2012; de Been et al., 2014; Alonso et al., 2017; Hussain et al., 2019; Ludden et al., 2019). Only two CMY-2 positive isolates belonged to phylogroup B2 and to ST131. The isolates from chickens in Hungary represented the B2-O25b-H4-ST131-*fimH*22-clade B lineage that is mostly composed of fluoroquinolone susceptible isolates, and has been associated with poultry and human bloodstream infections (Pitout and DeVinney, 2017). Several other STs found in our study, including ST88/C, ST93/A, ST101/B1, ST135/B2, ST617/A, ST648/F, and ST1431/B1, have frequently been determined in ESBL- and AmpC-producing *E. coli* from human and animal sources (Ewers et al., 2012; de Been et al., 2014; Pietsch et al., 2018; Schaufler et al., 2019; Apostolakos et al., 2020).

Thirty-one isolates revealed 10 of the sequence types (ST10, ST117, ST131, ST23, ST354, ST38, ST58, ST617, ST648, and ST88) that belong to the top 20 ExPEC STs, as previously determined based on a meta-analysis including 169 global studies (Manges et al., 2019). However, the classic ExPEC STs ST73 and ST95 that are successful extraintestinal pathogens but have also be reported as persistent intestinal colonizers in humans and animals were not present among our collection (Riley, 2014). It is commonly accepted that ExPEC lineages, such as ST95, ST73, ST12, and ST127 exhibit lower multidrug resistance levels, while only few are capable of combining MDR and virulence, like ST648, ST131, and ST410 (Ewers et al., 2014a, 2016; Schaufler et al., 2016; Manges et al., 2019).

The virulence potential of ESBL- and AmpC-producing *E. coli* originating from healthy livestock animals could be a significant issue for public health. Nevertheless, data on extensive virulence gene typing of AMR *E. coli* from livestock animals, including a rigorous screening for a set of > 800 VAGs related with intestinal and extraintestinal pathotypes, are scarce (Hussain et al., 2019; Apostolakos et al., 2020). We identified the intimin gene *eae*, which is a surrogate for atypical EPEC and several other InPEC-related genes, including the translocated intimin receptor gene *tir* in five ESBL- (SHV-12 and TEM-52) and one CMY-2-producing chicken *E. coli* isolates belonging to four different STs. Mueller et al. determined the *eae* gene in 3.6 and 2.9% of ESBL-producing *E. coli* isolates from livestock (*n* = 28) and healthy humans (*n* = 34), respectively. Consistent with our findings, they did not identify genes indicating the presence of EIEC (*ipaH*), ETEC (*sta, stb*, *est*), and STEC (*stx1, stx2*), suggesting a low prevalence of intestinal pathotypes among MDR bacteria from livestock (Mueller et al., 2016). Although bovines are the primary reservoir of STEC, previous reports confirm a low prevalence of ESBL-producing STEC among cattle and other livestock animals (Ewers et al., 2014b; Pietsch et al., 2018). Apostolakos et al. (2020) could assign the majority of 100 ESBL/pAmpC-*E. coli* isolated from the broiler production pyramid in Italy to defined ExPEC or InPEC pathotypes by virulence gene analysis. They identified 56% of their isolates as atypical EAEC, based on the presence of *aatA* and the absence of *aggR*. Both typical and atypical EAEC, that have been predominantly associated with pediatric diarrhea in developing countries, were not present in our isolates as they all lacked the pathotype-specific genes *aatA*, *aggR*, and *aaiC* (Bamidele et al., 2019).

Regarding ExPEC, distinct sequence types, particularly ST131, ST648, and ST410 have been reported as successful pandemic lineages that combine multidrug-resistance and virulence (Ewers et al., 2014a, 2016; Schaufler et al., 2016; Manges et al., 2019). Whether an *E. coli* isolate falls into the category of ExPEC, likely depends on the definition proposed by different authors. More than half of our 99 isolates fulfilled the criteria to be classified as avian ExPEC (APEC), including 19.6% isolates from non-avian sources. Apostolakos et al. (2020) identified 39 ESBL-*E. coli* broiler isolates (39%) belonging to 13 different STs as APEC. While they APEC group mainly (38.5%) consisted of ST744 and ST429/ST9298, our APEC strains were even more diverse (31 STs among 51 isolates) and only few STs were not unique, including ST117 (*n* = 6), ST23 (*n* = 4), ST38 (*n* = 3), ST88 (*n* = 3), and ST101 (*n* = 3). While we had no indication for a co-location of APEC-VAGs and ESBL-/AmpC genes on plasmids, others identified several virulence genes, including *sitA-D*, *iucA-D*, *iutA*, *hlyF*, *ompT*, *iss*, *iroN*, *cvaA-C*, and *cvi* on *bla*_CMY__–__2_ IncF plasmid obtained from a diseased French broiler, suggesting a putative threat for the easy dissemination of ExPEC virulence factors and resistance determinants (Touzain et al., 2018).

Only two of our isolates (CMY-2-ST38 and CTX-M-1-ST453) could be classified as ExPEC, whereas 67.7% of the isolates were considered as ExPEC-like (>10 ExPEC genes). Using the same criteria we applied, Apostolakos et al. (2020) identified 51% of their isolates belonging to 12 STs from different production chains of an integrated broiler company in Italy as ExPEC. In a study from Brazil, 58% of ESBL *E. coli* isolates from chicken carcasses harbored 3–5 of the ExPEC VAGs *iutA*, *hlyF*, *iss*, *iroN*, and *ompT* (Cyoia et al., 2018). The authors suggested that chicken meat is a potential reservoir of MDR *E. coli* strains harboring resistance and virulence genes that could pose a serious threat to human public health. This is further corroborated by a study dealing with ESBL-*E. coli* from poultry and human in India. Among 15 ExPEC-associated genes (*papA/C/E/F/G*, *fimH*, *pic*, *sat*, *tsh*, *vat*, *iutA*, *ireA*, *iroN*, *fyuA*, and *usp*) Hussain et al. (2019) identified comparable numbers in broiler (median 4) and human isolates (median 3) and overlaps in the AMR and VAG profile and phylogenetic background of a subset of strains.

Interestingly, several isolates harbored both InPEC- and ExPEC-related genes (Supplementary Document S1), resembling what has previously been reported as hybrid pathotype, which is probably best exemplified by EHEC as a long-standing EPEC/STEC hetero-pathogen (Santos et al., 2020). Our aEPEC strains additionally harbored 9–12 InPEC related genes, such as ColV plasmid gene *cvi*, iron acquisition gene *fyuA*, *iucA-D*, *irp2*, and *sitA-D*, increased serum survival protein gene *iss*, and haem acquisition protein gene *hma*, which plays a critical role in the colonization of the urinary tract. The ExPEC and APEC strains carried up to four InPEC-related genes, including those encoding for AidA-I adhesin-like protein invasion-associated protein Tia, long polar fimbriae Stg, serine protease Pic, iron-related haem receptor Iha, and ETEC F4 and F5 fimbriae. Apostolakos et al. (2020) identified 30 ESBL/AmpC broiler isolates that displayed an aEAEC/ExPEC pathotype. They suggested that the virulence gene repertoire of ESBL/pAmpC *E. coli* may explain their adaptation to and persistence in different niches. As the capacity for genome interrogations is constantly rising, it seems obvious that various VAGs, previously either linked with InPEC or ExPEC pathotypes, will be found among different *E. coli* pathotypes to which they have not traditionally been associated (Santos et al., 2020). This, together with the availability of extensive AMR data based on an ever growing number of genome sequences may help to predict the emergence of novel multidrug resistant and virulent strains.

## Conclusion

Our data suggest that cephalosporin resistance genes are mainly disseminated in livestock animals via distinct plasmids. Plasmid backbones within different plasmid lineages were often almost identical and were shared by phylogenetically unrelated isolates from chickens, cattle, and swine. In addition, they revealed significant similarity to plasmids from human isolates. We could demonstrate that *E. coli* of various phylogenetic groups and STs can combine antimicrobial resistance and virulence, even though a number of isolates could not be assigned to a distinct pathotype. In summary, this work significantly contributes to the understanding of the epidemiology and virulence potential of cephalosporin-resistant *E. coli* from livestock animals.

## Data Availability Statement

The datasets presented in this study can be found in online repositories. The names of the repository/repositories and accession number(s) can be found in the Supplementary Material.

## Ethics Statement

Ethical review and approval was not required for the study in accordance with the local legislation and institutional requirements.

## Author Contributions

CE supervised the entire project. CE and AJ drafted the manuscript. CE and FE designed the study. EP-B, CE, and AJ have provided raw data and analyzed the data. UL conducted the laboratory experiments. ST, CE, and TS conducted the analyses of the sequencing data. All authors critically reviewed the manuscript.

## Conflict of Interest

The authors declare that the research was conducted in the absence of any commercial or financial relationships that could be construed as a potential conflict of interest.

## Footnotes

1 https://cge.cbs.dtu.dk/services/ResFinder/

2 https://card.mcmaster.ca

3 https://cge.cbs.dtu.dk/services/MLST/

4 https://pubmlst.org/mlst/

5 https://cge.cbs.dtu.dk/services/SerotypeFinder/

6 https://cge.cbs.dtu.dk/services/FimTyper/

7 https://enterobase.warwick.ac.uk/

8 https://pubmlst.org/plasmid/

## Abbreviations

AMR: antimicrobial resistance
APEC: avian pathogenic *E. coli*
CARD: Comprehensive Antibiotic Resistance Database
CGE: Center for Genomic Epidemiology
EHEC: enterohemorrhagic *E. coli*
EIEC: enteroinvasive *E. coli*
EPEC: enteropathogenic *E. coli*
ESC: extended-spectrum cephalosporin
ExPEC: extraintestinal pathogenic *E. coli*
InPEC: intestinal pathogenic *E. coli*
MALDI-TOF MS: matrix-assisted laser desorption/ionization- time of flight mass spectrometry
MLST: multilocus sequence typing
pMLST: plasmid multilocus sequence typing
ST: sequence type
UPEC: uropathogenic *E. coli*
VAG: virulence-associated gene
WGS: whole genome sequencing.
