# Supplementary material for: Genomic Diversity and Virulence Potential of ESBL- and AmpC-β-Lactamase-Producing Escherichia coli Strains From Healthy Food Animals Across Europe
Source: Front Microbiol. 2021 Apr 1;12:626774. doi: 10.3389/fmicb.2021.626774 (PMC8047082; doi:10.3389/fmicb.2021.626774)
Supplement: Supplementary file 1 [file Data_Sheet_1.zip › Supplementary Material Folder/Supplementary Document_S3.pdf]

Supplementary Document S3

Figure S3: Circular representation of CTX-M-1 IncIα/pST-3 plasmids from 21 *E. coli* isolates of this study compared with reference plasmid pC60-108 (GenBank: KJ484635.1; chicken, Switzerland, 2013).

Figure S4: Circular representation of CTX-M-1 IncN/pST-1 plasmids from two *E. coli* isolates of this study compared with reference plasmid pL2-43 (GenBank: KJ484641.1; lamb, Switzerland, 2013

Figure S5: Circular representation of CTX-M-14 plasmids from four *E. coli* isolates from this study and of chromosomal fragments of isolates IHIT32077 and IHIT32078 carrying *bla*CTX-M-14 on the chromosome compared with reference plasmid CMY-2 2016C-3936C1 unnamed2 (GenBank: CP018772; *E. coli*, human, USA).

Figure S6: Circular representation of SHV-12 IncI1α (pST-3, pST-26, and pST-95) plasmids from 16 *E. coli* isolates of this study compared with reference plasmid SHV-12 IncI1α/pST-95 pCAZ590 (GenBank: LT669764.1; chicken, Germany, 2011).

Figure S7: Circular representation of SHV-12 IncX3 plasmids from four *E. coli* isolates of this study compared with reference plasmid pEC-244 (GenBank: KX618704; chicken feces).

Figure S8: Circular representation of TEM-52 IncI1α pST-36/CC-3 plasmids from three *E. coli* isolates of this study compared with reference plasmid pESBL-117 (GenBank: CP008734.1; human urine, the Netherlands).

Figure S9: Circular representation of CMY-2 IncI1α/pST-12 plasmids from 10 *E. coli* isolates of this study compared with reference plasmid p11-004736-1-7_99 (GenBank: NZ_CP016516; *Salmonella* Heidelberg, Canada, 2011).

Figure S10: Circular representation of CMY-2 IncI1α/p-ST2 plasmids from two *E. coli* isolates of this study compared with reference plasmid pSA01AB09084001_92 (GenBank: NZ_CP016533.1; *Salmonella* Heidelberg, chicken cecal content, Canada, 2009).

Figure S11: Circular representation of CMY-2 IncK2 plasmids from seven *E. coli* isolates of this study compared with reference plasmid pDV45 (GenBank KR905384.1; poultry retail meat).

Figure S12: Circular representation of CMY-2 IncA/C/pST-3 plasmid from one *E. coli* isolate of this study compared with reference plasmids pKP-Goe_024-3 GenBank: NZ_CP018704.1; human, abdominal fluid, Germany, 2014) and pSH163_135 (GenBank: JN983045; Turkey, diagnostic specimen, USA, 2002).

Figure S13: Circular representation of CMY-2 plasmids with unknown Inc type from two *E. coli* isolates of this study compared with reference plasmid 2016C-3936C1 unnamed2 (GenBank: CP018772; *E. coli*, human, USA).

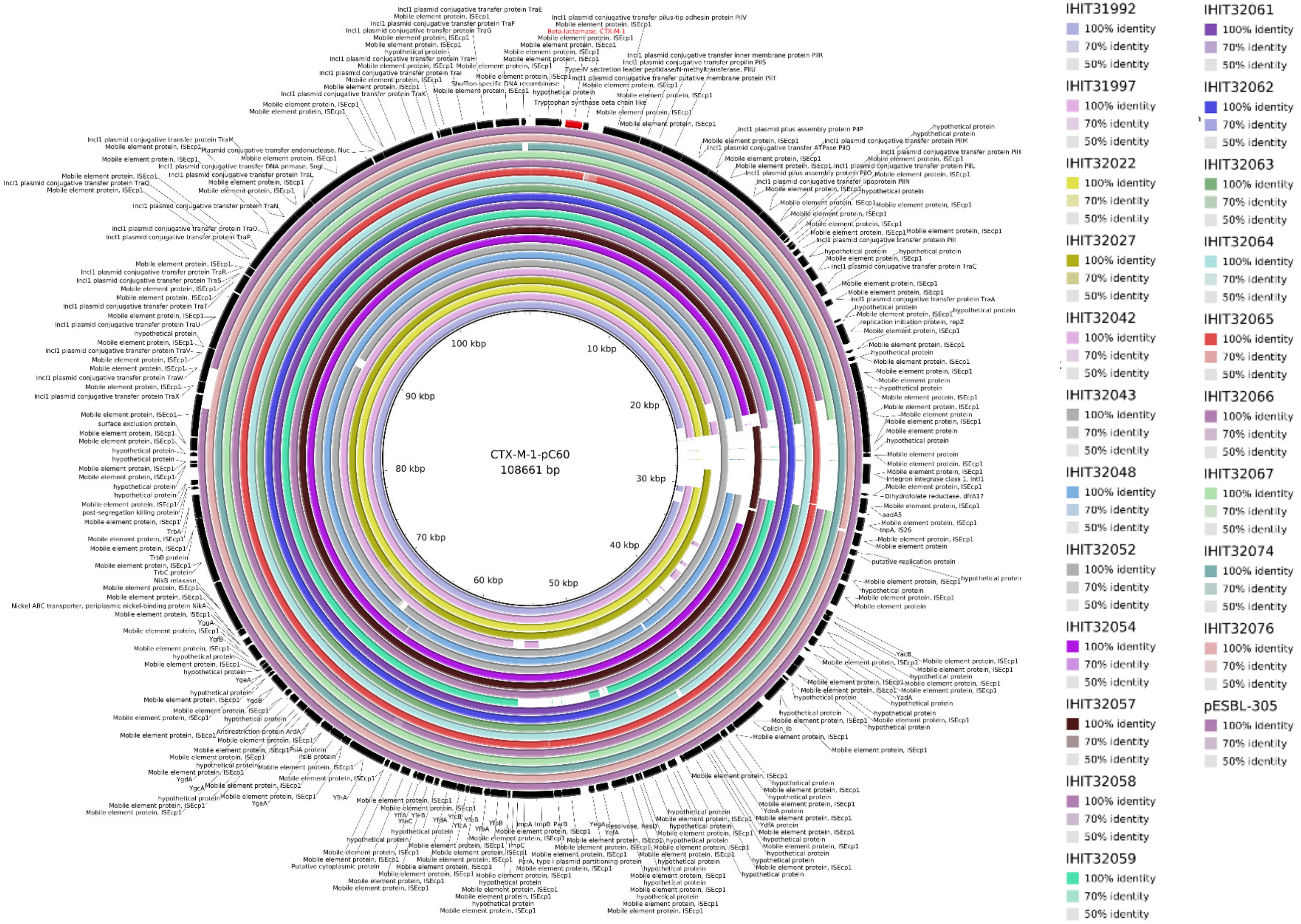

Figure S3: Circular representation of CTX-M-1 IncIα/pST-3 plasmids from 21 *E. coli* isolates of this study compared with reference plasmid pC60-108 (GenBank: KJ484635.1; chicken, Switzerland, 2013). The second last circle represents plasmid pESBL-305 (GenBank: CP008737.1) that also reveals high similarity to pC60 and to our presumed plasmids. The outermost circle shows the coding sequence of the reference plasmid; red, *bla*$_{\text{CTX-M-1}}$ gene.

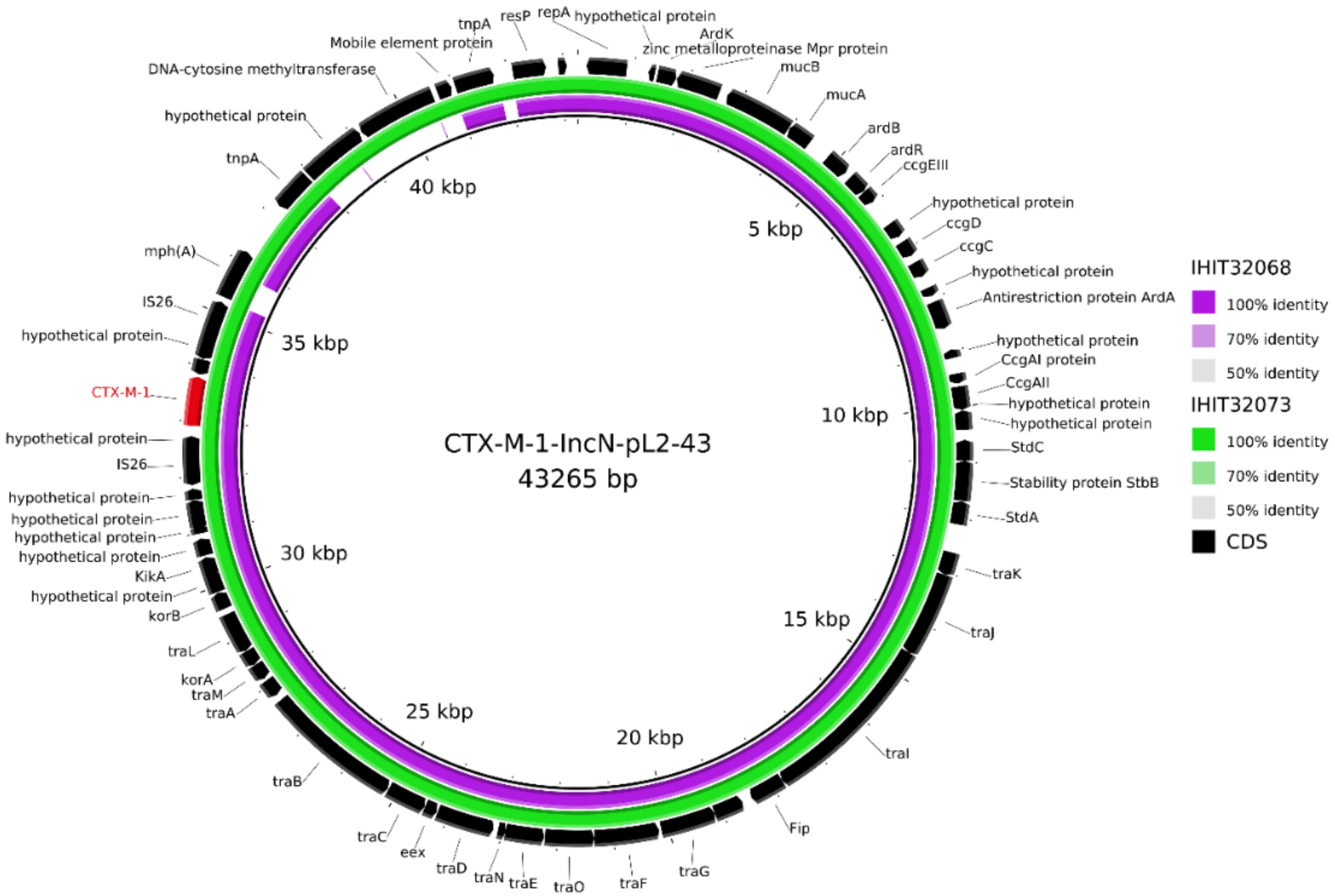

Figure S4: Circular representation of CTX-M-1 IncN/pST-1 plasmids from two *E. coli* isolates of this study compared with reference plasmid pL2-43 (GenBank: KJ484641.1; lamb, Switzerland, 2013). The outermost circle shows the coding sequence of the reference plasmid; red, *bla*$_{CTX-M-1}$ gene.

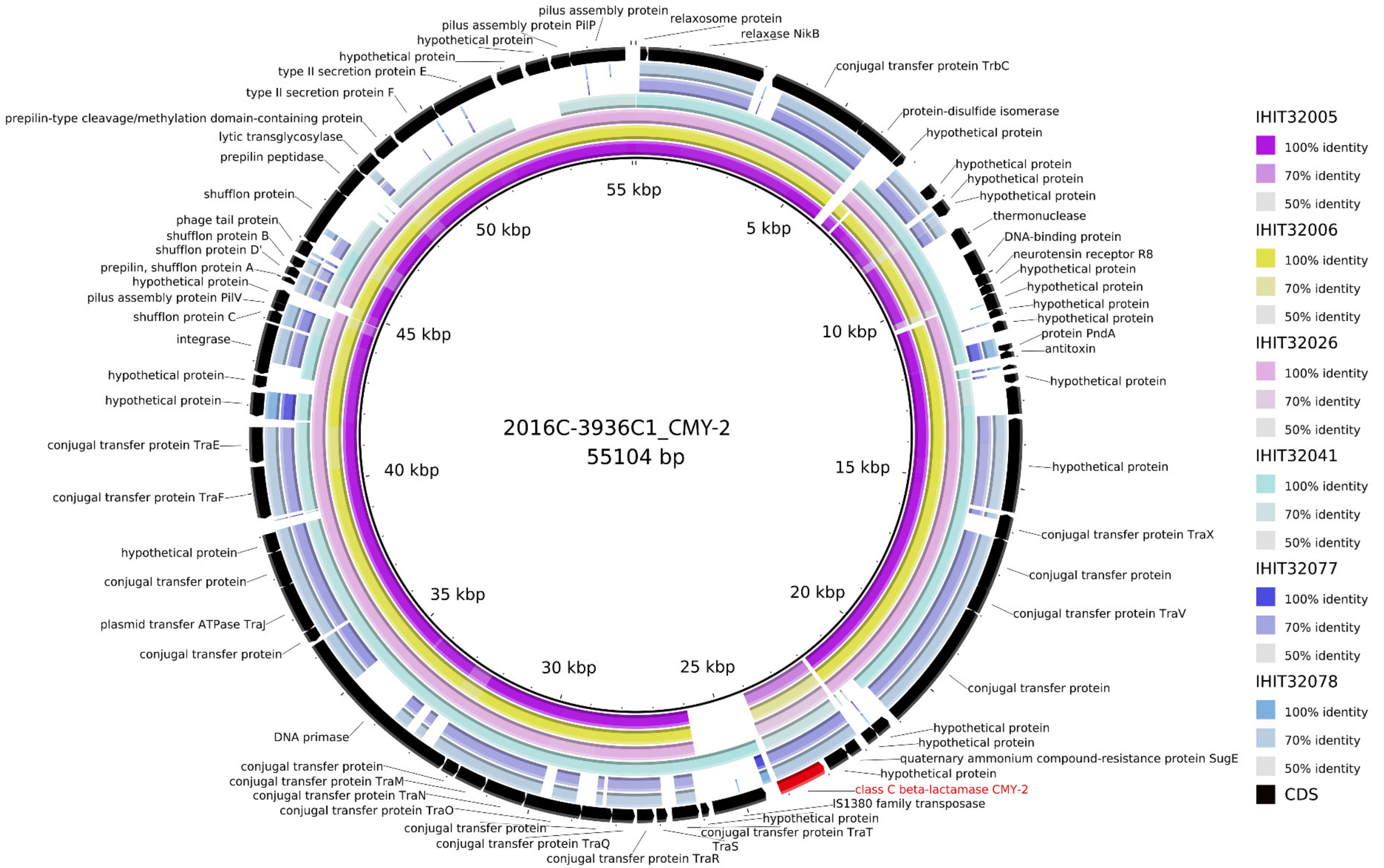

Figure S5: Circular representation of CTX-M-14 plasmids from four *E. coli* isolates from this study and of chromosomal fragments of isolates IHIT32077 and IHIT32078 carrying *bla*CTX-M-14 on the chromosome compared with reference plasmid CMY-2 2016C-3936C1 unnamed2 (GenBank: CP018772; *E. coli*, human, USA). The outermost circle shows the coding sequence of the reference plasmid; red, *bla*$_{CMY-2}$ gene.

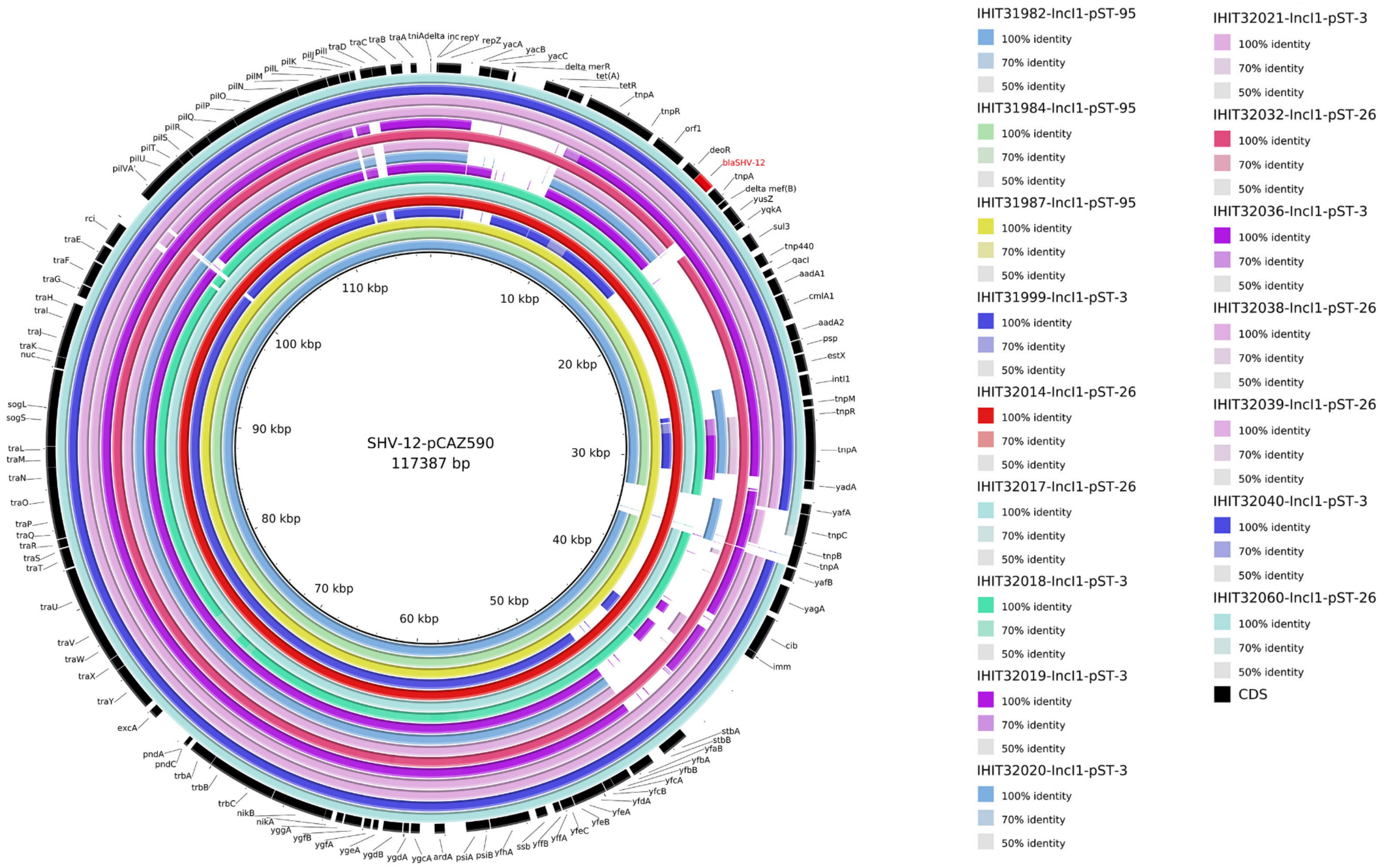

Figure S6: Circular representation of SHV-12 IncI1α (pST-3, pST-26, and pST-95) plasmids from 16 *E. coli* isolates of this study compared with reference plasmid SHV-12 IncI1α/pST-95 pCAZ590 (GenBank: LT669764.1; chicken, Germany, 2011). The outermost circle shows the coding sequence of the reference plasmid; red, *bla*$_{\text{SHV-12}}$ gene.

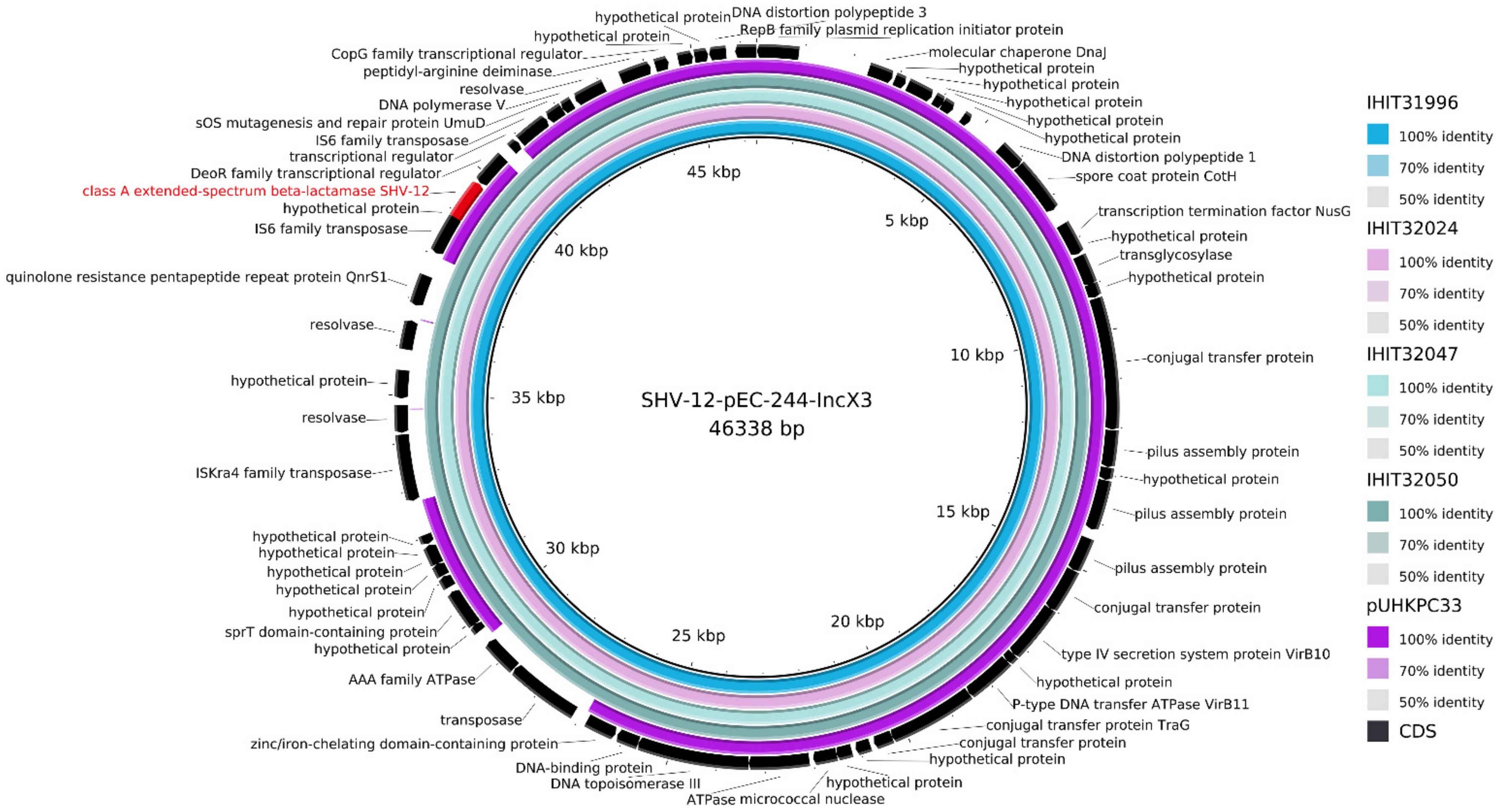

Figure S7: Circular representation of SHV-12 IncX3 plasmids from four *E. coli* isolates of this study compared with reference plasmid pEC-244 (GenBank: KX618704; chicken feces). For further comparison, plasmid pUHKPC33 (GenBank NZ_CP011992), a 43.48 kb SHV-12 IncX3 plasmid from a human *K. pneumoniae* isolate was aligned to the reference plasmid. The outermost circle shows the coding sequence of the reference plasmid; red, *bla*$_{SHV-12}$ gene.

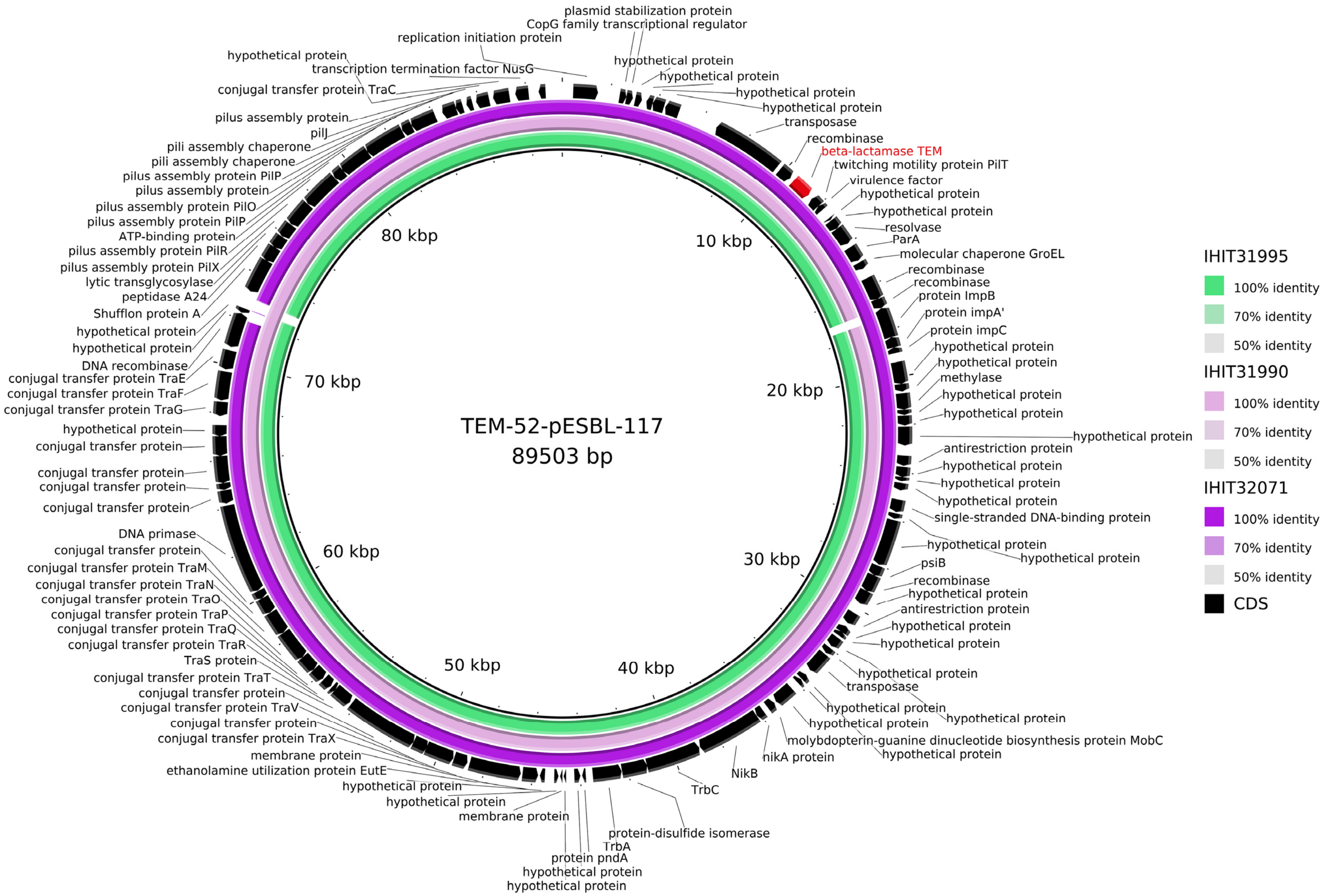

Figure S8: Circular representation of TEM-52 IncI1α pST-36/CC-3 plasmids from three *E. coli* isolates of this study compared with reference plasmid pESBL-117 (GenBank: CP008734.1; human urine, the Netherlands). The outermost circle shows the coding sequence of the reference plasmid; red, *bla*$_{\text{TEM-52}}$ gene.

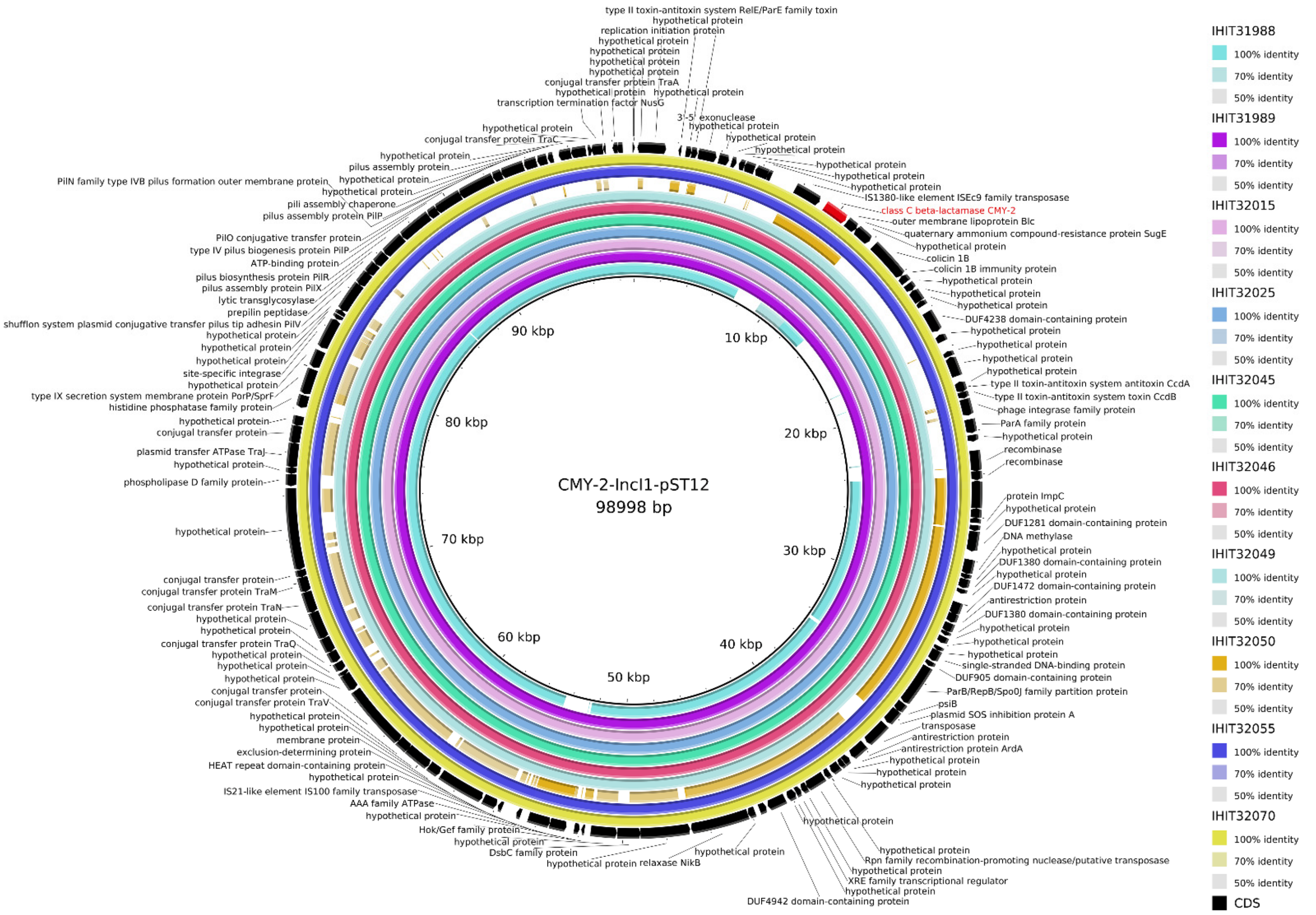

Figure S9: Circular representation of CMY-2 IncI1α/pST-12 plasmids from 10 *E. coli* isolates of this study compared with reference plasmid p11-004736-1-7_99 (GenBank: NZ_CP016516; *Salmonella* Heidelberg, Canada, 2011). The outermost circle shows the coding sequence of the reference plasmid; red, *bla*$_{CMY-2}$ gene.

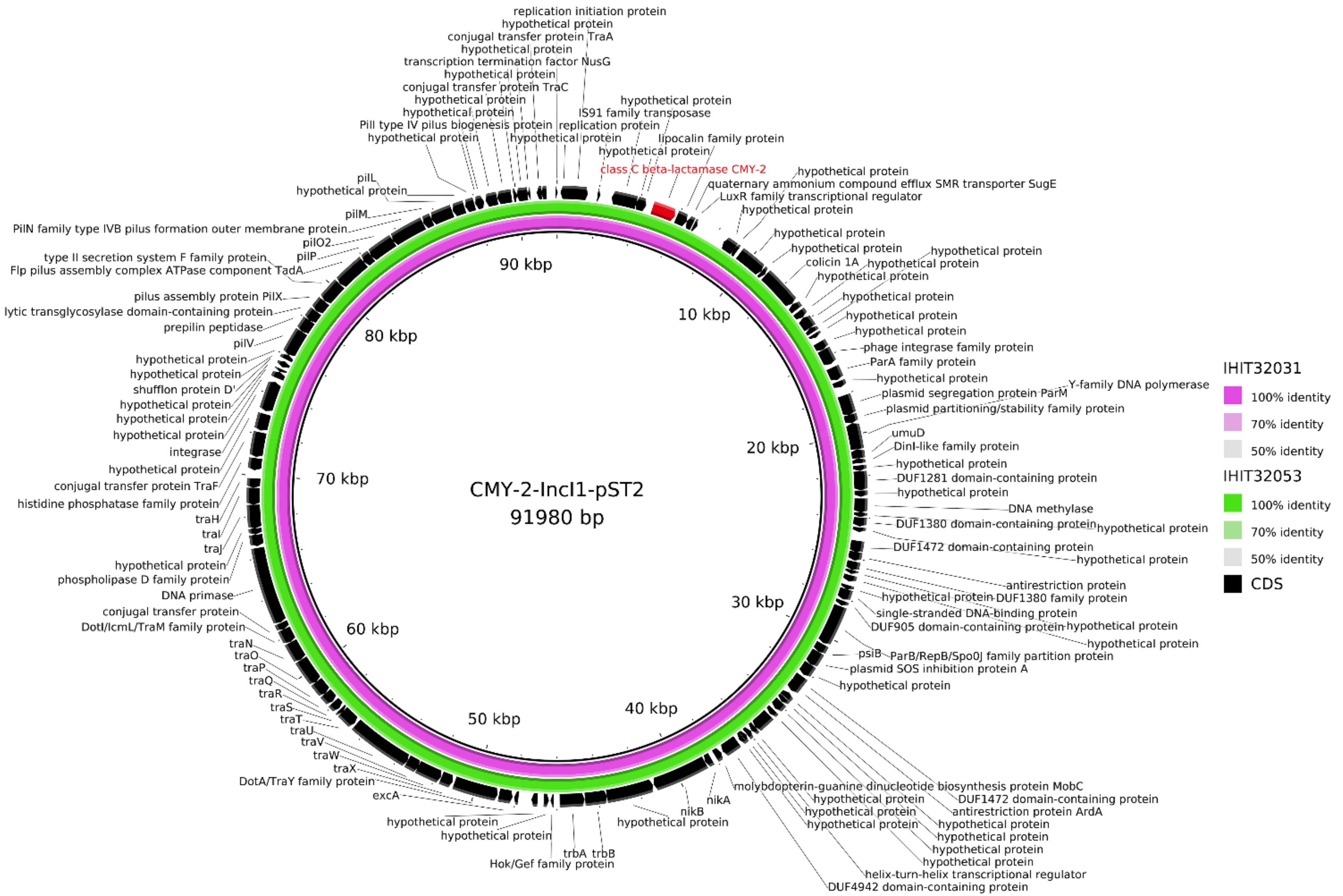

Figure S10: Circular representation of CMY-2 IncI1α/p-ST2 plasmids from two *E. coli* isolates of this study compared with reference plasmid pSA01AB09084001_92 (GenBank: NZ_CP016533.1; *Salmonella* Heidelberg, chicken cecal content, Canada, 2009). The outermost circle shows the coding sequence of the reference plasmid; red, *bla*$_{CMY-2}$ gene.

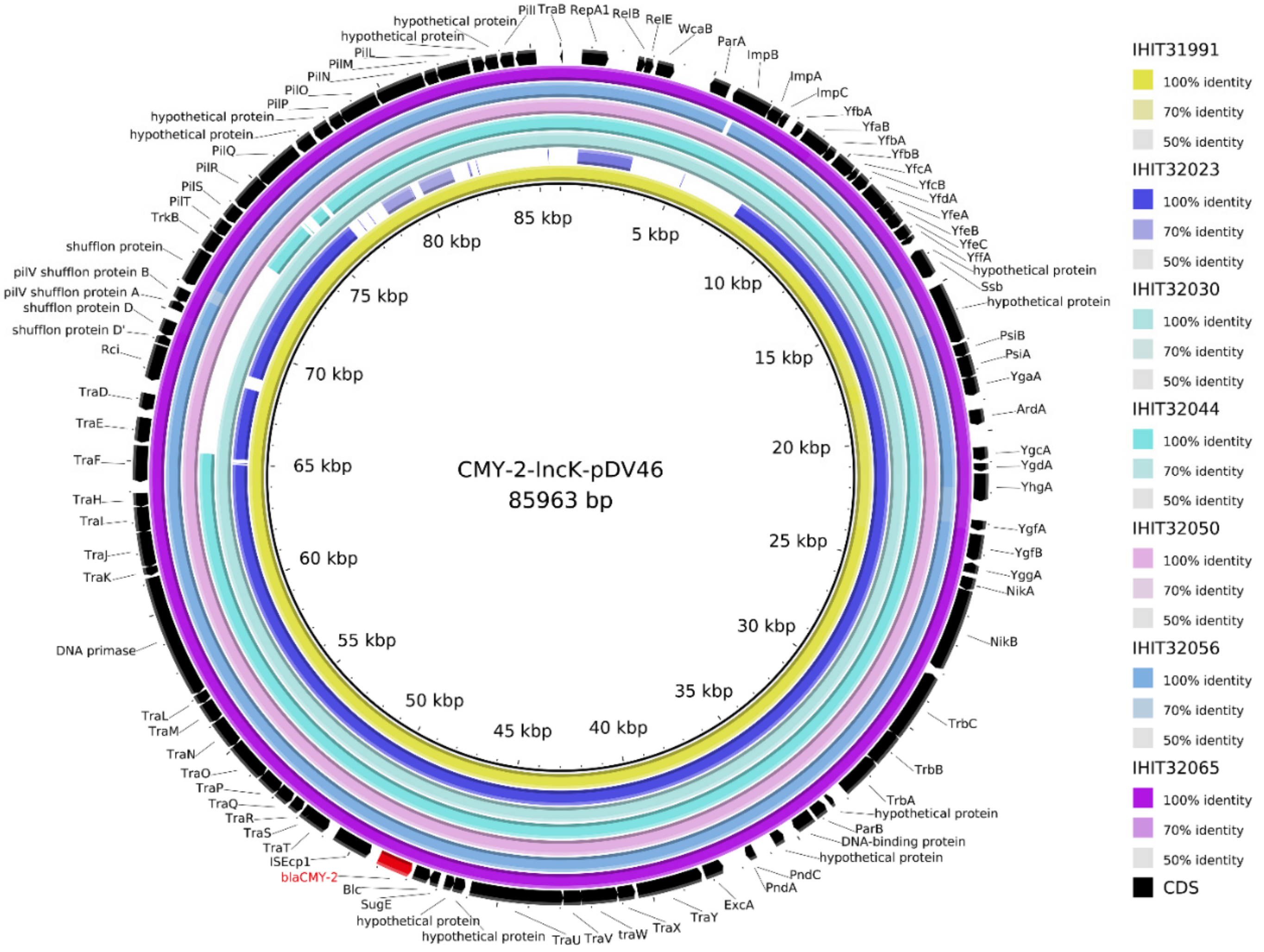

Figure S11: Circular representation of CMY-2 IncK2 plasmids from seven *E. coli* isolates of this study compared with reference plasmid pDV45 (GenBank KR905384.1; poultry retail meat). The outermost circle shows the coding sequence of the reference plasmid; red, *bla*$_{\text{CMY-2}}$ gene.

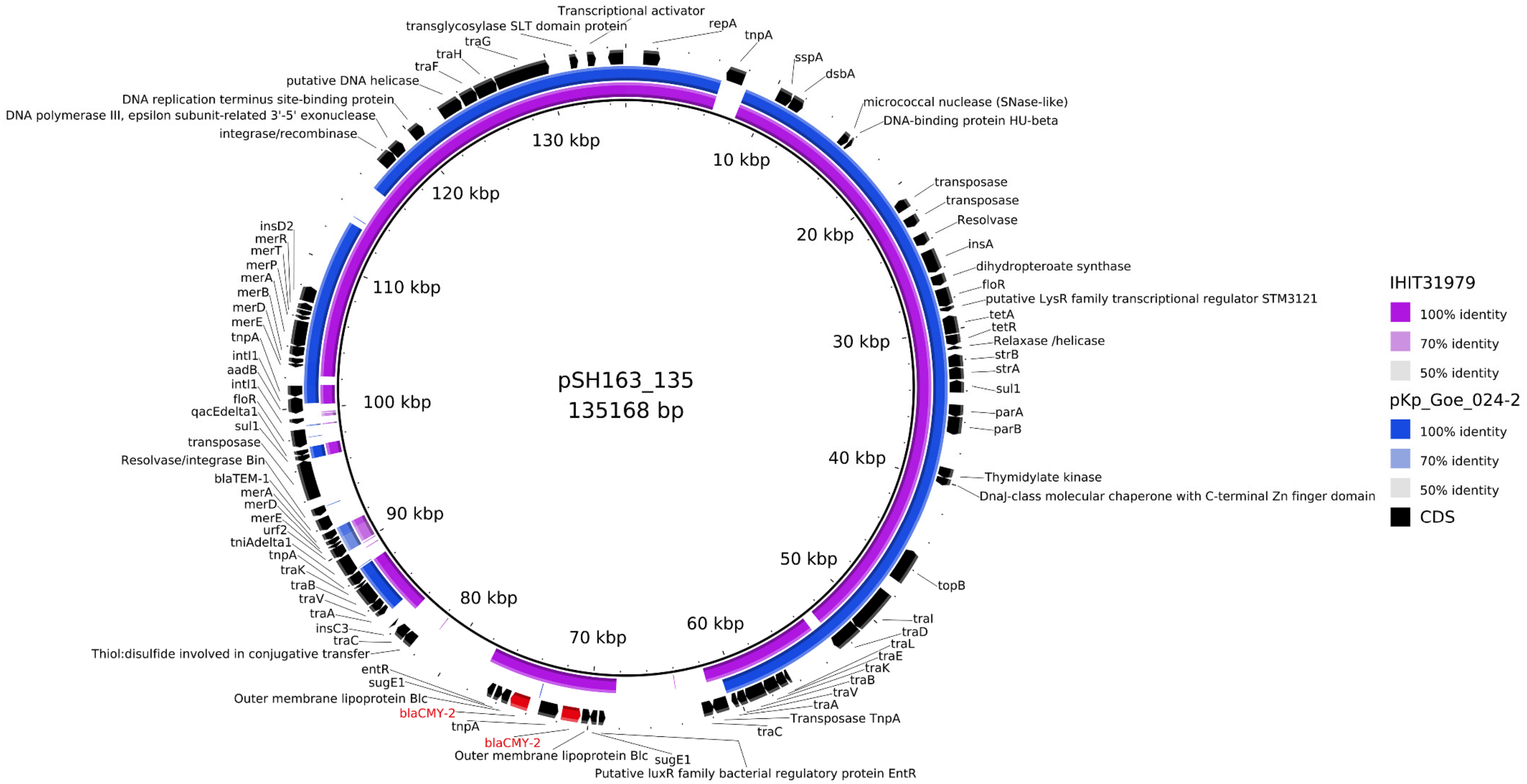

Figure S12: Circular representation of CMY-2 IncA/C/pST-3 plasmid from one *E. coli* isolate of this study compared with reference plasmids pKP-Goe_024-3 GenBank: NZ_CP018704.1; human, abdominal fluid, Germany, 2014) and pSH163_135 (GenBank: JN983045; Turkey, diagnostic specimen, USA, 2002). The outermost circle shows the coding sequence of the reference plasmid; red, *bla*$_{\text{CMY-2}}$ gene.

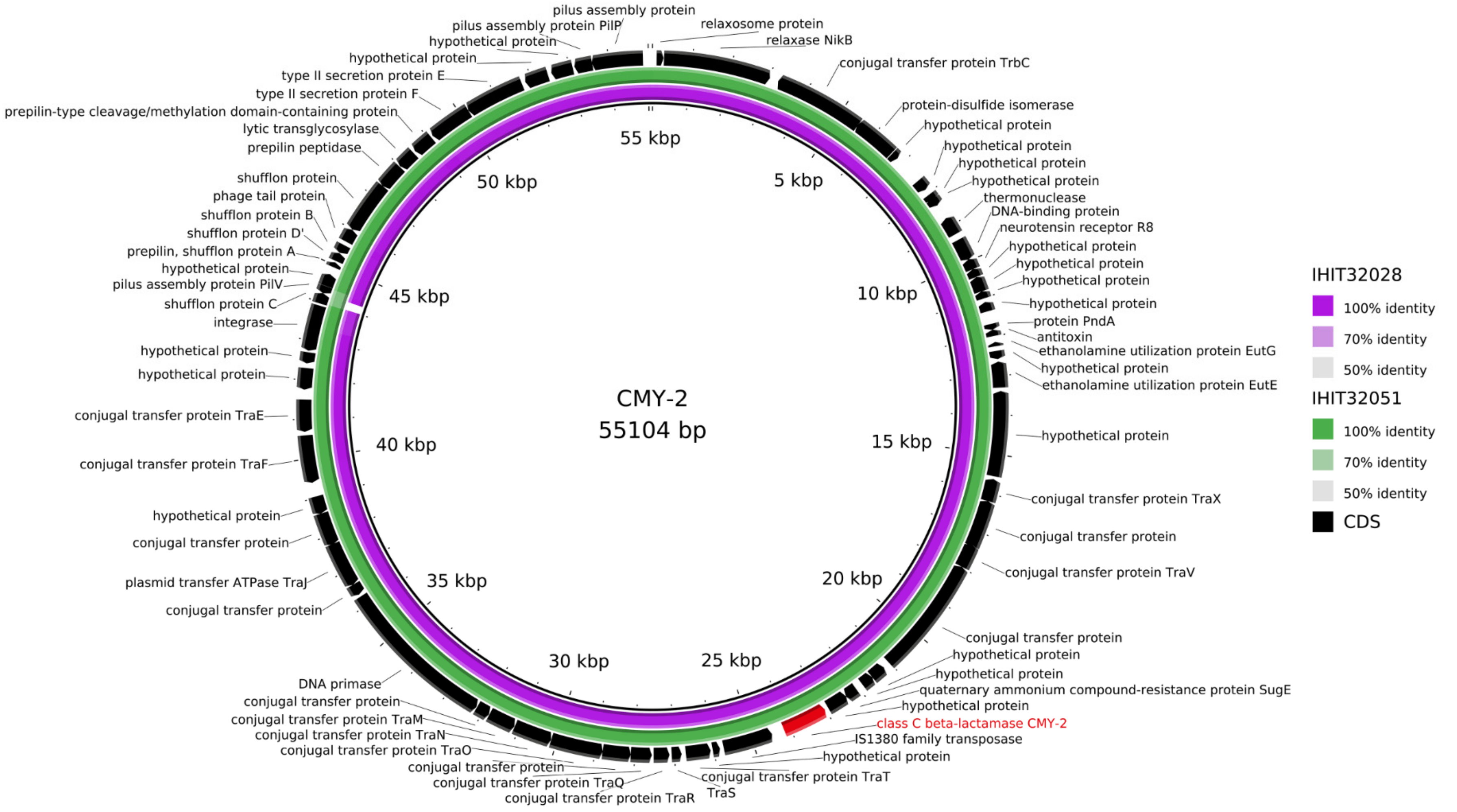

Figure S13: Circular representation of CMY-2 plasmids with unknown Inc type from two *E. coli* isolates of this study compared with reference plasmid 2016C-3936C1 unnamed2 (GenBank: CP018772; *E. coli*, human, USA). The outermost circle shows the coding sequence of the reference plasmid; red, *bla*$_{CMY-2}$ gene.
