# Supplementary material for: Genomic Diversity and Virulence Potential of ESBL- and AmpC-β-Lactamase-Producing Escherichia coli Strains From Healthy Food Animals Across Europe
Source: Front Microbiol. 2021 Apr 1;12:626774. doi: 10.3389/fmicb.2021.626774 (PMC8047082; doi:10.3389/fmicb.2021.626774)
Supplement: Supplementary file 1 [file Data_Sheet_1.zip › Supplementary Material Folder/Supplementary Figure S2.pdf]

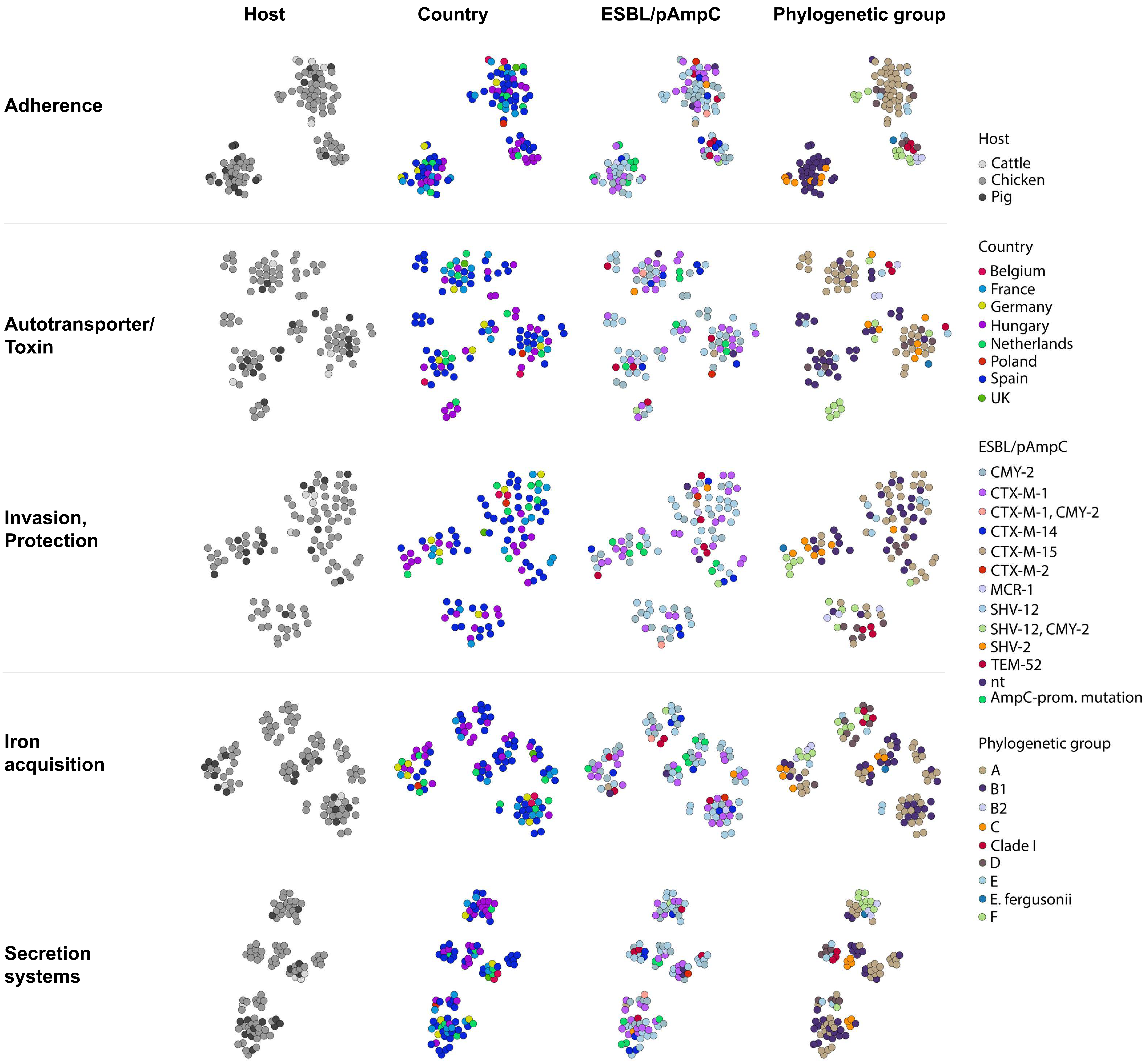

FIGURE S2 | Distribution of Virulence-associated genes among 99 ESC-non-S/ESC-R *Escherichia* spp. isolates with respect to host, country, ESBL/pAmpC type and phylogenetic group
