# Supplementary material for: Genomic Diversity and Virulence Potential of ESBL- and AmpC-β-Lactamase-Producing Escherichia coli Strains From Healthy Food Animals Across Europe
Source: Front Microbiol. 2021 Apr 1;12:626774. doi: 10.3389/fmicb.2021.626774 (PMC8047082; doi:10.3389/fmicb.2021.626774)
Supplement: Supplementary file 1 [file Data_Sheet_1.zip › Supplementary Material Folder/Supplementary Tables S1-S4.pdf]

Supplementary Tables

1 Supplementary Tables

TABLE S1:
Characteristic features of reference plasmids used for alignment of WGS contigs and for sequence comparison

TABLE S2:
AMR patterns (n=34) among 99 *E. coli* and one *E. fergusonii* isolate from livestock animals

TABLE S3:
Distribution of 49 ExPEC-related genes/gene clusters among 99 *Escherichia* spp. isolates

TABLE S4
Distribution of virulence associated genes (VAGs) among 99 *Escherichia* spp. strains with respect to phylogenetic group, host, and extended-spectrum cephalosporin resistance determinant

TABLE S1 | Characteristic features of reference plasmids used for alignment of WGS contigs and for sequence comparison

Plasmid public database	Inc group	pMLST	ESBL/ pAmpC	Spezies	Source	Size	GenBank	Reference
pDV45	IncK2	nt	CMY-2	*E. coli*	Poultry, retail meat	85963	KR905384.1	(Seiffert et al., 2017)
p11-004736-1-7_99	IncI1	ST12/-	CYM-2	*S.* Heidelberg	Bovine, organ, Canada, 2011	98998	NZ_CP016516	(Labbe et al., 2016)
2016C-3936C1 plasmid unnamed2	unknown	nt	CYM-2	E. coli	Human, USA, O157	55104	CP018772	(Lindsey et al., 2017)
pSA01AB0908400 1_92	IncI1	ST2/CC-2	CMY-2	*S.* Heidelberg	Chicken, cecal content, Canada, 2009	91980	NZ_CP016533.1	(Labbe et al., 2016)
pSH163_135	IncA/C	ST3/-	CMY-2	*S.* Heidelberg	Turkey, diagnostic specimen, Ohio, 2002	135168	JN983045	(Han et al., 2012)
pC60-108	IncI1	ST3/CC-3	CTX-M-1	E. coli	Chicken, Switzerland, 2013	108661	KJ484635.1	(Wang et al., 2014)
pL2-43	IncN	ST1/-	CTX-M-1	*E. coli*	Lamb, Switzerland, 2013	43265	KJ484641.1	(Wang et al., 2014)
pESBL-305	IncI1	ST3/CC-3	CTX-M-1	*E. coli*	Chicken caecum content, Netherlands	107552	CP008737.1	(Brouwer et al., 2014)
pUHKPC33	IncX3	nt	SHV-12	*K. pneumoniae*	Human (hospital), USA	43380	NZ_CP011992	(Wright et al., 2014)
pEC-244	IncX3	nt	SHV-12	*E. coli*	Chicken faeces	46338	KX618704	(Liakopoulos et al., 2018)
pCAZ590	IncI1	ST95/CC-9	SHV-12	*E. coli*	Chicken, Germany, 2011	117387	LT669764.1	(Alonso et al., 2017)
pESBL-117	IncI1	ST36/CC-3	TEM-52	*E. coli*	Human urine, Netherlands	89503	CP008734.1	(Brouwer et al., 2014)
pDKX-TEM-52*	IncX1	nt	TEM-52	*E. coli*	Chicken meat, Denmark, 2006	38611	JQ269336.1	(Johnson et al., 2012)
pKP_Goe_024-2	IncA/C	ST3/-	none	*K. pneumoniae*	Human, abdominal fluid, Germany, 2014	96073	NZ_CP018704.1	direct submission
pMCR-1-CT*	IncX4	nt	MCR-1	*E. coli*	Human, O157:H48, USA	33304	CP018773.2	(Lindsey et al., 2017)

* asterisk indicates that plasmids have only been described in the main text and are not included in BRIG figures.

References to TABLE S1

Alonso, C.A., Michael, G.B., Li, J., Somalo, S., Simon, C., Wang, Y., et al. (2017). Analysis of *bla*$_{SHV-12}$-carrying *Escherichia coli* clones and plasmids from human, animal and food sources. *J Antimicrob Chemother* 72(6), 1589-1596. doi: 10.1093/jac/dkx024.

Brouwer, M.S., Bossers, A., Harders, F., van Essen-Zandbergen, A., Mevius, D.J., and Smith, H.E. (2014). Complete Genome Sequences of IncI1 Plasmids Carrying Extended-Spectrum beta-Lactamase Genes. *Genome Announc* 2(4). doi: 10.1128/genomeA.00859-14.

Han, J., Lynne, A.M., David, D.E., Tang, H., Xu, J., Nayak, R., et al. (2012). DNA sequence analysis of plasmids from multidrug resistant *Salmonella enterica* serotype Heidelberg isolates. *PLoS One* 7(12), e51160. doi: 10.1371/journal.pone.0051160.

Johnson, T.J., Bielak, E.M., Fortini, D., Hansen, L.H., Hasman, H., Debroy, C., et al. (2012). Expansion of the IncX plasmid family for improved identification and typing of novel plasmids in drug-resistant Enterobacteriaceae. *Plasmid* 68(1), 43-50. doi: 10.1016/j.plasmid.2012.03.001.

Labbe, G., Ziebell, K., Bekal, S., Macdonald, K.A., Parmley, E.J., Agunos, A., et al. (2016). Complete Genome Sequences of 17 Canadian Isolates of Salmonella enterica subsp. enterica Serovar Heidelberg from Human, Animal, and Food Sources. *Genome Announc* 4(5). doi: 10.1128/genomeA.00990-16.

Liakopoulos, A., van der Goot, J., Bossers, A., Betts, J., Brouwer, M.S.M., Kant, A., et al. (2018). Genomic and functional characterisation of IncX3 plasmids encoding *bla*$_{SHV-12}$ in *Escherichia coli* from human and animal origin. *Sci Rep* 8(1), 7674. doi: 10.1038/s41598-018-26073-5.

Lindsey, R.L., Batra, D., Rowe, L., Loparev, V.N., Stripling, D., Garcia-Toledo, L., et al. (2017). High-Quality Genome Sequence of an *Escherichia coli* O157 Strain Carrying an *mcr-1* Resistance Gene Isolated from a Patient in the United States. *Genome Announc* 5(11). doi: 10.1128/genomeA.01725-16.

Seiffert, S.N., Carattoli, A., Schwendener, S., Collaud, A., Endimiani, A., and Perreten, V. (2017). Plasmids Carrying *bla*$_{CMY-2/4}$ in *Escherichia coli* from Poultry, Poultry Meat, and Humans Belong to a Novel IncK Subgroup Designated IncK2. *Front Microbiol* 8, 407. doi: 10.3389/fmicb.2017.00407.

Wang, J., Stephan, R., Power, K., Yan, Q., Hachler, H., and Fanning, S. (2014). Nucleotide sequences of 16 transmissible plasmids identified in nine multidrug-resistant *Escherichia coli* isolates expressing an ESBL phenotype isolated from food-producing animals and healthy humans. *J Antimicrob Chemother* 69(10), 2658-2668. doi: 10.1093/jac/dku206.

Wright, M.S., Perez, F., Brinkac, L., Jacobs, M.R., Kaye, K., Cober, E., et al. (2014). Population structure of KPC-producing *Klebsiella pneumoniae* isolates from midwestern U.S. hospitals. *Antimicrob Agents Chemother* 58(8), 4961-4965. doi: 10.1128/AAC.00125-14.

TABLE S2 | AMR patterns (n=34) among 99 *E. coli* and one *E. fergusonii* isolate from livestock animals

Antimicrobial resistance pattern	No. of isolates	No. of antibiotic classes
PEN-ESC-CEPH-FQ-PHEN-TET-SULF-FOLATE	3	8
PEN-ESC-AMINO-FQ-TET-SULF-FOLATE	1	7
PEN-ESC-CEPH-FQ-TET-SULF-FOLATE	1	7
PEN-ESC-CEPH-PHEN-TET-SULF-FOLATE	1	7
PEN-ESC-FQ-PHEN-TET-SULF-FOLATE	4	7
PEN-ESC-AMINO-FQ-TET-SULF	2	6
PEN-ESC-CEPH-AMINO-TET-SULF	1	6
PEN-ESC-CEPH-TET-SULF-FOLATE	2	6
PEN-ESC-FQ-PHEN-TET-SULF	5	6
PEN-ESC-FQ-TET-SULF-FOLATE	5	6
PEN-ESC-PHEN-TET-SULF-FOLATE	3	6
PEN-ESC-PMB-TET-SULF-FOLATE	1	6
PEN-ESC-CEPH-FQ-TET	1	5
PEN-ESC-CEPH-SULF-FOLATE	1	5
PEN-ESC-CEPH-TET-SULF	2	5
PEN-ESC-FQ-TET-SULF	2	5
PEN-ESC-PHEN-TET-SULF	8	5
PEN-ESC-TET-SULF-FOLATE	8	5
PEN-PHEN-TET-SULF-FOLATE	1	5
PEN-ESC-CEPH-FQ	1	4
PEN-ESC-CEPH-SULF	3	4
PEN-ESC-CEPH-TET	4	4
PEN-ESC-FQ-SULF	2	4
PEN-ESC-FQ-TET	2	4
PEN-ESC-PHEN-SULF	2	4
PEN-ESC-SULF-FOLATE	6	4
PEN-ESC-TET-SULF	7	4
PEN-ESC-CEPH	7	3
PEN-ESC-FQ	2	3
PEN-ESC-SULF	5	3
PEN-ESC-TET	3	3
PEN-TET-SULF	1	3
PEN-ESC	2	2
PEN-FQ	1	2

ESC, extended-spectrum cephalosporins (cefotaxime and/or ceftazidime); PEN, penicillins; CEPH, cephamycins; AMINO, aminoglycosides; FQ, fluorquinolones; PHEN, phenicols; TET, tetracyclines; SULF, sulphonamides; FOLATE, folate pathway inhibitors.

TABLE S3 | Distribution of 49 ExPEC-related genes/gene clusters among 99 *Escherichia* spp. isolates

Virulence factors and categories	Gene(s)	% positive isolates
**Adhesion**		
Afimbrial adhesin	*afaABCDEFG*	0
Antigen 43	*agn43*	6.1
APEC autotransporter adhesin	*aatA*	0
Curli fimbriae	*csgABCDEFG*	97.0
Dr antigen-specific adhesin	*draABCDE*	0
ExPEC Adhesin I	*yqi*	1.0
F1C fimbriae	*focG*	0
Iron regulated outer membrane protein	*ireA*	11.1
P fimbriae	*papABCDEFGHIJK*	4.0
S fimbriae	*sfaABCDEFGH*	0
Temperature sensitive haemagglutinin	*tsh*	17.2
Type 1 fimbriae	*fimABCDEFGHI*	93.9
UPEC trimeric autotransporter adhesin	*upaC*	2.0
**Invasion-related factors**		
Invasion of brain epithelium	*ibeA*	6.1
Genetic island of meningitis	*gimB*	0
**Iron uptake**		
Aerobactin siderophore system	*iucABDC*	54.5
Aerobactin siderophore receptor	*iutA*	1.0
E. coli iron transport	*eitABC*	21.1
Enterobactin siderophore	*entABCDEFS*	98.0
Iron repressible proteins	*irp1/irp2*	22.2/23.2
Iron transport system	*sitABCD*	66.7
Salmochelin siderophore system	*iroBCDEN*	46.5
Outer membrane hemin receptor	*chuA*	30.3
**Protectin/Serum resistance**		
Transfer protein	*traT*	75.8
K1 capsular polysaccharide	*neu*	4.0
Group II capsular antigen	*kpMTII*	21.2
Outer membrane protease	*ompT*	48.5
Increased serum survival protein	*iss*	77.8
Colicin V	*cvaC/cvi*	27.3/46.5
Colicin B	*cba*	9.1
ColicinE	*celb*	11.1
Colicin M	*cma*	29.3
Microcin H47	*mchF*	21.2
**Toxins/Hemolysins**		
Secreted autotransporter toxin	*sat*	0
Polyketide synthase	*pks*	0
Colibactin synthesis	*clbA-R*	0

**Virulence factors and categories**	**Gene(s)**	**% positive isolates**
Vacuolating autotransporter toxin	*vat*	3.0
Hemolysin A	*hlyA*	1.0
Hemolysin F	*hlyF*	53.5
Cytotoxic necrotizing factors	*cnf1/cnf2/cnf3*	0
Heat-stable enterotoxin	*EAST-1*	31.3
**Miscellaneous/Protease/Autotransporter**		
PAI marker	*malX*	22.2
Serin protease autotransporter	*pic*	6.1
Uropathogenic-specific protein	*usp*	8.1
ExPEC secreted metalloprotease	*yghJ*	72.7
Autotransporter protein UpaC	*upaC*	2.0
E. coli transport system	*etsAB*	48.5

TABLE S4 | Distribution of virulence associated genes (VAGs) among 99 *Escherichia* spp. strains with respect to phylogenetic group, host, and extended-spectrum cephalosporin resistance determinant

Category	Mean number of VAGs* ± standard deviation
**Phylogenetic group**	
A (n=34)	102.6 ± 14.0
B1 (n=28)	113.9 ± 13.3
B2 (n=3)	119.7 ± 2.7
C (n=7)	127.1 ± 14.9
Clade I (n=3)	113.0 ± 2.0
D (n=8)	134.4 ± 13.7
E (n=5)	130.8 ± 14.1
F (n=10)	115.6 ± 12.1
E. fergusonii (n=1)	47.0 ± 0
**Host**	
Chicken (n=80)	113.5 ± 14.9
Pig (n=15)	117.0 ± 17.5
Cattle (n=4)	89.5 ± 9.6
**Resistance determinant**	
CMY-2 (n=22)	119.5 ± 16.7
CTX-M-1 (n=25)	114.5 ± 22.0
CTX-M-2 (n=1)	84.0 ± 0
CTX-M-14 (n=6)	99.2 ± 16.7
CTX-M-15 (n=1)	104.0 ± 0
SHV-2 (n=1)	113.0 ± 0
SHV-12 (n=32)	111.4 ± 10.1
TEM-52 (n=5)	113.0 ± 6.0
AmpC promoter mutation (n=7)	125.2 ± 11.6
MCR-1 (n=1)	94.0 ± 0
unclear (n=2)	89.0 ± 0
